# Comparison of Different Fruit Shapes of *Lanxangia tsaoko*: Differences and Relevance of Phenotypes, Antioxidant Activities, and Physicochemical Information

**DOI:** 10.1002/fsn3.71993

**Published:** 2026-06-30

**Authors:** Dengke Fu, Tianmei Yang, Weize Yang, Zongliang Xu, Meiquan Yang, Yuanzhong Wang, Jinyu Zhang

**Affiliations:** ^1^ Medicinal Plants Research Institute, Yunnan Academy of Agricultural Sciences Kunming China; ^2^ College of Traditional Chinese Medicine, Yunnan University of Chinese Medicine Kunming Yunnan China

**Keywords:** biological activity, *Lanxangia tsaoko*, metabolic profiles, SVM

## Abstract

There is a wide variety of *Lanxangia tsaoko* (LT) fruit shapes, and there has been no systematic characterization of the chemical composition of the fruit shapes and their functions. In this study, a comprehensive comparison of the three fruit shapes of LT was carried out from the macroscopic to the microscopic level. The results showed that the redder and longer LT had a stronger aroma and that the formation of aroma was closely related to flavonoids and phenolic acids. In addition, flavonoids and their synthesis pathways were the main reasons for the differences among the three fruit shapes. In terms of flavor, the ROAV values of 2‐nonenal, (*E*)‐2‐octenal, (*E*)‐2‐dodecenal, and (*E*)‐ were much higher than those of the other volatiles, and they were the most dominant aroma components in LT. After that, a support vector machine model based on fourier transforms near infrared spectroscopy was developed for fast identification of different fruit types of LT. The best performance of the model was achieved after second‐order derivative preprocessing with 100% correctness. The final correlation analysis illustrated the interpretability between metabolites and spectral features in LT and provided theoretical guidance for the development of non‐destructive, efficient, and low‐cost techniques.

## Graphical abstract

This study selected three fruit types of LT, including PCS, ES, and LFS. We examined the phenotypes and physicochemical components, volatile substances, non‐volatile substances, and FT‐NIRS characteristics of the three fruit types. Based on FT‐NIRS, an SVM model was established for the rapid identification of LT fruit types. Additionally, we conducted multiple correlation analyses in the text to explore potential correlations between phenotypes, between volatile and non‐volatile substances, between antioxidant capacity and metabolites, and between metabolites and spectral characteristic values.

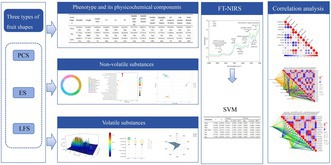

## 1 Introduction

*Lanxangia tsaoko* (LT) which is mainly distributed in southeast Asian countries such as China, Vietnam, and India, is one of the important spice substances in the world (Li et al. 2021; Liang et al. 2023; Yang et al. 2022). As food, LT removes fishy odor, increases appetite, and has a lemon‐like flavor (Liang et al. 2024). As herbal medicine, based on essential oils, flavonoids, phenolic acids, diphenylheptane, and other active ingredients, it confers antimicrobial, anti‐inflammatory, antidiabetic, neuroprotective, gastrointestinal tract regulating, antitumor, and other pharmacological activities (Fan et al. 2023; Yang et al. 2023; Pu 2022; Yang et al. 2022). Among them, essential oils have been most widely studied and applied in several fields, such as cosmetics, food, agriculture, and drug manufacturing (Cui et al. 2017). However, numerous factors influence the quality of LT, spanning from cultivation and management to production and processing. For example, the large altitude difference in the growing environment and the wide regional distribution lead to significant variations of LT in spatial location. Studies have shown that the biomass of LT is positively related to the altitude, and there are also differences in chemical information between different origins in the regions of Yunnan province such as Pingbian, Jinping, Maguan, Dulongjiang, and Tengchong (Li et al. 2021).

It is worth mentioning that fruit shapes, as an important agronomic trait in crops, have a significant impact on the commercial value and consumer preference of LT. According to the pharmacopeia of the people's republic of China (2020 edition), the fruit shapes of LT are oval. However, researchers have found that this is not a single fruit shape in the field in Yunnan, and the fruit is classified into elliptic shape, cone shape, spindle shape, and spheroidal shape based on the aspect ratio of the fruit (Li et al. 2021). The formation of LT fruit shapes is inevitably affected by genetic mechanisms and environmental factors, but there is no report on the regulation of fruit shapes properties (Liang et al. 2023). However, the results of the current study have illustrated that environmental factors have important effects on LT fruit shapes. First, there is a positive correlation between altitude and LT biomass, which suggests that fruit size is larger at higher altitudes (Li et al. 2021). Second, most of the regions possessed the four classical fruit shapes, except for a few regions. For example, there were no fusiform fruits in Gongshan, and there were fewer fusiform fruit shapes in Luchun. In addition, the highest fruit shapes diversity index was found in Pingshan and the lowest in Gongshan (Li et al. 2021). This may be related to the main ecological factors affecting the growth of LT, such as temperature, altitude, and precipitation (He, Shi, et al. 2024; He, Yang, and Wang 2024). The coefficients of variation for seed, seed number, and fruit weight were large (23.84%–26.82%) among the different fruit shapes of LT, while the long axis length and short axis length were relatively stable (Wei et al. 2019). Phenotypic variation among different fruit shapes was variable within a certain region, and overall, the differences between populations were greater than within populations (Li et al. 2021; Wei et al. 2019). It has been shown that there are some differences in volatile content and major volatiles among fruit shapes. Of these, (*E*)‐2‐hexenal, octanal, α‐phellandrene, α‐terpinene, cymene, (*E*)‐2‐octenal, and γ‐terpinene were positively correlated with fruit length. Trans‐sabinenehydrate was negatively correlated with fruit length (Li et al. 2021). In addition, the popular folk belief that fruit length is positively correlated with aroma concentration has also been experimentally demonstrated (Li et al. 2021).

In the present study, we analyzed a wide range of terpenes and aldehydes based on ultra performance liquid chromatography mass spectrometry‐ mass spectrometry (UPLC/MS–MS) for broadly targeted metabolomics and two‐dimensional gas chromatography–mass spectrometry (GC × GC‐TOFMS) full two‐dimensional mass spectrometry flavoromics were used to comprehensively analyze the nonvolatile and volatile substances in different fruit shapes of LT. With the help of broadly targeted metabolomics, we investigated the attenuation of bitterness in the fermentation process of black garlic and found that non‐volatiles might contribute to the ester aroma substances through correlation analysis. Substances may contribute to the production of ester‐like aroma substances (Huang 2024). The full two‐dimensional approach of GC × GC overcomes the 1DGC in separating highly complex mixtures or resolving highly overlapping compounds byanalysis speed by combining two columns that are independent of each other in an orthogonal manner. It achieves high resolution, high separation capacity, and high peak capacity, and has a strong viability in the detection of complex flavor components such as origin differentiation, wine identification, and liquor vintage identification (Li, Mo, et al. 2024; Li, Zhao, et al. 2024; Li, Zhu, et al. 2024). In plant‐derived flavor research, this technology has been utilized to analyze the aroma‐active compounds in Zanthoxylum essential oil, successfully identifying trace key aroma components such as thioethers and terpenes, and clarifying their contribution to the numbing‐spicy flavor (Ma et al. 2026). Similarly, in fermented foods, GC × GC‐TOFMS detected over 40% more volatile substances compared to one‐dimensional GC‐TOFMS and accurately identified flavor components—such as organic acids, esters, and heterocyclic compounds—that synergize with taste (including umami), providing technical support for elucidating the “sour‐umami” complementary mechanism (Kallio et al. 2006; Paiva et al. 2025). Fourier transforms near infrared spectroscopy (FT‐NIRS) is a fast, nondestructive green and easy‐to‐analyze way to identify food products by obtaining O—H, N—H, C—H, CH_2_, C=C—H, CH_2_, C=C, and C=H, etc., and obtains the full chemical information of the sample within seconds (Chen et al. 2023). Currently, FT‐NIRS is commonly used for rapid prediction of compositional content and rapid identification of quality. For example, the content in porcini mushrooms was rapidly and accurately predicted by FT‐NIRS with a regression model, thus solving the problems of high cost and complex instrumentation of traditional methods (Deng et al. 2024). In the study of fruits, vegetables, and spices, researchers have successfully utilized FT‐NIRS to achieve rapid and simultaneous prediction of organic acids, free amino acids, phenolic compounds, and key flavor components (Deng et al. 2025; Fu et al. 2026). In substrates such as edible fungi, medicinal plants, and spices, this technology can quickly model the content changes of umami amino acids and taste‐active organic acids, effectively distinguishing differences in taste quality among different harvest periods, developmental stages, and treatment groups (Deng et al. 2025; Xia et al. 2024). Additionally, FT‐NIRS can integrate sensory evaluation data to establish correlation models between spectral information and sensory attributes such as umami, sourness, and richness, enabling indirect and rapid prediction of taste and flavor. This provides an efficient technical solution for monitoring the dynamic accumulation of flavor compounds, analyzing taste interactions, and rapid grading of agricultural product flavor quality (Chapman et al. 2019; Dong et al. 2019).

Previous studies focused on the correlations between fruit shape and ecological factors, as well as phenotypic variations, but lacked evidence at the metabolic level. The main objective of this study is to reveal the differences in metabolic levels among various fruit shapes, and to clarify the intrinsic relationship between fruit phenotypic differences and metabolites. This study focuses on fruits of different morphological materials, conducting multidimensional quality analysis and rapid discrimination research, demonstrating significant innovation. The research systematically integrates antioxidant activity, nutritional components, volatile aroma compounds, and non‐volatile flavor substances, breaking through the limitations of single quality indicators to comprehensively reveal the differential characteristics of physiological and flavor qualities in fruits of varying morphologies. It clarifies the accumulation patterns and quality correlations of multiple components. Moreover, this study innovatively combines FT‐NIRS spectroscopy technology with SVM machine learning models to establish a non‐destructive and efficient fruit classification and identification method. Compared to traditional detection and classification approaches, it significantly simplifies experimental procedures and enhances detection efficiency. By integrating material basis with rapid detection techniques, this research provides theoretical support and technical references for fruit resource utilization, quality evaluation, and rapid traceability classification.

## 2 Materials and Methods

### 2.1 Sample Collection

LT samples were collected from three collection sites belonging to Yunnan and Guangxi, and the fruits of three LT plants were collected from each collection site using the “Z” sampling method. All the samples in this study were identified as LT by Zhang Jinyu, a botanist from the Institute of Medicinal Plants of Yunnan Academy of Agricultural Sciences. Based on the fruit aspect ratio and phenotypic observation, the collected fruits were classified into three varieties, namely pine cone shape (PCS), long fruit shape (LFS), and ellipse shape (ES), and the specific locations are shown in Table S1. The morphologic characteristics of PCS fruits are that they have a large longitudinal diameter and a significant difference between the two ends, and one end is sharp while the other is round. The morphology is characterized by having a large longitudinal diameter and a significant difference between the two ends, with one end being sharp and the other rounded. The LFS morphology is characterized by having a large aspect ratio and sharp ends. The ES morphology is characterized by having a long longitudinal diameter and rounded ends (Li et al. 2021; Wei et al. 2019). As shown in Figure S1, the biggest difference between the three fruit types is the morphology of the ends. The LT fruits are harvested in October during the ripening period. Only those without pest or disease infections and with a light red color are selected. For each fruit shape, 10 fruits are chosen. The three fruit shapes were left to dry naturally at room temperature, after which they were pulverized, sieved through a 100‐mesh sieve, and stored in a −4°C refrigerator for later use.

### 2.2 Determination of Phenotype, Physiological Parameters, and Antioxidant Capacity

The length and width of the fruit shapes were measured by vernier calipers (model: SL01‐1, measuring range: 0–150 mm, resolution: 0.1 mm, manufacturer: Deqing Shengqinxin Electronic Technology Co. Ltd.). The dry weights were measured by an electronic balance (model: 30002, specification: Max = 3000 g, manufacturer: Huizhou Yingheng Electronic Technology Co. Ltd.). Comparison of colors involved a Ci6X spectrophotometer (Acuity Brands Ltd., Shanghai, China), with a light source of D65/10, and the three values of L*, a*, and b* were measured. L* stands for black and white, with larger values representing whiter (brighter) and smaller values representing blacker (darker); a* stands for red and green, with larger values representing redder and smaller values representing greener; b* stands for yellow and blue, with larger values representing yellower and smaller values representing bluer, represents the more blue (Li, Mo, et al. 2024; Li, Zhao, et al. 2024; Li, Zhu, et al. 2024). In addition, soluble sugar, soluble protein, soluble solids, total phenol, titratable acid, ash, were determined by DNS colorimetric method, BCA colorimetric method, refractometer method, forintol colorimetric method, acid–base titration method, and direct ashing method, respectively (Fu et al. 2025; Park et al. 2025; Ramírez‐Brewer et al. 2024; Yu et al. 2025). The antioxidant power of fruit shapes was determined by purchasing kits (Nanjing Morfan Biotechnology Co. Ltd.), 1,1‐diphenyl‐2‐picryl‐hydrazyl radical (DPPH), ferric ion reducing antioxidant power (FRAP), 2,2′‐Azinobis‐ 3‐ethylbenzthiazoline‐6‐sulphonate (ABTS) activity. The test procedure was carried out in strict accordance with the instructions. For specific instructions, please refer to the Appendix S1.

### 2.3 E‐Nose Detection

Volatile components were determined using a PEN3 electronic nose (AIRSENSE, Germany). Before the test, 2.00 g of LT samples were weighed into 200 mL glass bottles and sealed with plastic wrap, and the containers were allowed to stand at 25°C for 30 min to stabilize the gas state. The sampling time interval was set at 1 s/group, the sensor self‐cleaning time was 120 s, the zeroing time was 5 s, the sample preparation time was 5 s, the inlet flow rate was 400 mL/min, and the analysis time of the experiment was 150 s (Wang, Gao, et al. 2024; Wang, Wang, et al. 2024; Wang, Xie, et al. 2024). Ten different metal oxide sensors were used as the sensing columns in PEN3. Their sensitivity preferences for substances were W1C, aromatic components; W5S, small molecule nitrogen oxides; W3C, ammonia and aromatic components; W6S, hydrides; W5C, short‐chain alkane aromatics; W1S, methane; W1W, sulfides; W2S, ethanol; W2W, aromatic components and organosulfides; and W3S, alkanes. In addition, we chose the 147th, 148th, and 149th s with stable performance as the time of data collection according to Figure S2.

### 2.4 Broadly Targeted Metabolomics

The methanol, acetonitrile, and formic acid used in the experimental procedure were of chromatographically pure grade. In addition, the brands of methanol and acetonitrile were Merck, and the brand of formic acid was Aladdin.

#### 2.4.1 Sample Extraction, Chromatography, and Mass Spectrometry Conditions

First, 50 mg of the three fruit shapes sample powders were weighed precisely using an electronic balance (MS105DΜ) and added to 70% methanol aqueous internal standard extract (1200 μL—pre‐cooled at 20°C, less than 50 mg was added at the rate of 1200 μL of extractant per 50 mg of sample). Vortexing was performed at 30‐min intervals, vortexing 6 times, each lasting 30 s. Afterwards, the samples were centrifuged at 12,000 rpm for 3 min, and the supernatant was taken, filtered through a microporous filter membrane (0.22 μm pore size), and stored in the injection bottle. The information was collected by ultra performance liquid chromatography (UPLC) and tandem mass spectrometry (MS/MS). The column was an Agilent SB‐C18 1.8 μm, 2.1 mm × 100 mm, with ultrapure water (with 0.1% formic acid added) as phase A and acetonitrile (with 0.1% formic acid added) as phase B. The B‐phase ratio was 5% for 0.00 min, and then the B‐phase ratio was increased linearly to 95% within 9 min and maintained for 1 min, and decreased again to 95% for 1 min. A flow rate of 0.35 mL/min, a column temperature of 40°C and an injection volume of 2 μL were maintained for the above procedure. An electrospray ionization (ESI) ion source was used in the mass spectrometry at 550°C with an ion spray voltage of 5500 V (positive ion mode)/−4500 V (negative ion mode). The ion source gas I, gas II, and curtain gas were set at 50, 60, and 25 psi, respectively, and the collision‐induced ionization parameter was set to high. Triple quadrupole mass spectrometer (QQQ) scans using multiple reaction monitoring (MRM) mode with collision gas (nitrogen) set to medium. Further optimization was done by declustering potential (DP) and collision energy (CE) to complete the DP and CE of individual MRM ion pairs. Finally, a specific set of MRM ion pairs were monitored in each period based on the metabolites eluted within each period (Fu et al. 2025).

#### 2.4.2 Qualitative and Quantitative

The characterization is based on the metware database built by Maiwei combined with secondary mass spectrometry information. Isotopic signals, repetitive signals containing K^+^ ions, Na^+^ ions, NH4^+^ ions, and repetitive signals of fragment ions that are themselves other larger molecular weight substances were removed from the analysis. After obtaining the metabolite mass spectrometry data from different samples by MRM mode, peak area integration was performed on the chromatographic peaks of all metabolites, and the integration was corrected for the mass spectrometry peaks of the same metabolite in different samples among them.

### 2.5 Flavouromics

#### 2.5.1 Preparation of Internal Standard Solution

Appropriate amount of deuterated n‐hexanol‐d13 standard was pipetted, and 50% aqueous ethanol solution was added to make a 1 mg/L master standard solution, which was stored in a refrigerator at 4°C. In addition, an appropriate amount of 1000 mg/L n‐alkane standard was pipetted and diluted step by step with n‐hexane to form a 1 mg/L solution, which was stored in a refrigerator at 4°C.

#### 2.5.2 Flavor Extraction

Take an appropriate amount of sample in a 20 mL headspace vial; add 10 μL of the ISTD solution to each sample. Incubate the sample at 80°C for 10 min; put the SPME fiber in the chamber at 270°C for 10 min before extracting the sample. Transfer the SPME to the incubator at 80°C for 25 min; desorb the SPME fiber at 250°C for 5 min in the GC injector. Put the SPME fiber in the chamber at 270°C for 10 min after the injection step. Transfer 10 μL of the n‐alkanes into a 20 mL headspace vial; incubation extraction and injection (Li 2022).

#### 2.5.3 GC × GC Analysis

Analyses were carried out using a LECO Pegasus 4D instrument (LECO, St. Joseph, MI, USA) consisting of an Agilent 8890A GC (Agilent Technologies, Palo Alto, CA, USA) system equipped with a split/splitless injector, and dual stage cryogenic modulator (LECO) coupled with TOFMS detector (LECO). A DB‐Heavy Wax (30 m × 250 μm I.D., 0.5 μm) (Agilent, USA) was used as first dimension column (1D) and Rxi‐5Sil MS (2.0 m × 150 μm I.D., 0.15 μm) (Restek, USA) was used as a second‐dimension column (2D). The carrier gas was high purity helium (> 99.999%) at a constant flow rate of 1.0 mL/min. The temperature program of the oven was as followed: the oven temperature was held at 50°C for 2 min at first, then raised to 230°C at the rate of 5°C/min and held for 5 min. The secondary oven temperature was operated at 5°C higher than the first oven. The temperature of the modulator is always 15°C higher than that of the second column. The modulator was operated with a 6 s modulation period. The GC injector temperature was 250°C (Li 2022).

#### 2.5.4 MS Conditions

Flavor substance was performed on LECO Pegasus BT 4D. The transfer line and TOF MS ion source temperature were set at 250°C and 250°C, respectively. The acquisition frequency was 200 spectra/s. The mass spectrometer was operated in the EI mode at 70 eV using a range of m/z 35–550 and the detector voltage of 1960 V (Li 2022).

#### 2.5.5 Calculation of ROAV

(1) $$\text{r}{\mathrm{OAV}}_{i}=C_{i}/{\mathrm{OT}}_{i}$$

(2) $$\text{ROAVi}={\text{rOAV}}_{i}/{\text{rOAV}}_{\max}\times 100$$

First, rOAV was determined by the ratio of the relative amount of each compound (*C*_*i*_) to its threshold in water (OT_*i*_) obtained from the reference and Odor database. ROAVs were calculated by the ratio of rOAVs of a volatile (rOAV_*i*_) to the maximum rOAVs among the corresponding sample (rOAV_max_) (Fu et al. 2026).

### 2.6 FT‐NIRS Acquisition and Preprocessing

With NIRS (Thermo Fisher Scientific INC., USA), we obtained spectral data for the three fruit shapes. Drawing on the experience of He, Shi, et al. (2024), He, Yang, and Wang (2024), we performed the following operations. First, the instrument was started and warmed up for 2 h, and the sample information was collected when the instrument state was at a steady value, and the experimental humidity and temperature were kept near 45% and 25°C, respectively, during the experiment. Ten dry fruits of similar size and color were selected for spectral collection. The instrument was used in diffuse reflectance mode; both background and spectral scans were 64 times with a resolution of 4 cm^−1^, wavenumber range of 10,000–4000 cm^−1^. Each sample was scanned twice and averaged for subsequent analysis. In addition, background information was collected for spectral correction every 1 h of spectral data collection to allow moisture and air to interfere with the spectral data. The average spectra were obtained in OMNIC 8.0 and then entered SIMAC 14.1 to collect the spectral data matrix. Finally, we used multiplicative scatter correction (MSC), standard normal variate (SNV), first‐order derivative (1st), second‐order derivative (2nd), and their groups as spectral preprocessing methods to improve spectral resolution, eliminate random noise, and enhance variability among spectral data (He, Shi, et al. 2024; He, Yang, and Wang 2024).

### 2.7 Support Vector Machine (SVM)

We constructed a qualitative SVM model based on the FT‐NIRS data of different fruit shapes to enable a rapid identification of the fruit shapes of LT. As a supervised learning model, SVM is widely employed for the classification and regression analysis of datasets. In SVM, “c” and “g” are two crucial parameters. Where *c* is the penalty coefficient, and with a large value of *c*, the model tends to overfit more; however, with a small value of *c*, the model also tends to underfit. Whether it is too large or too small, it will lead to a decrease in the performance of the SVM model. However, *g* determines the distribution of the data mapped to the new feature space. The number of support vectors affects the training and prediction speed of the model; however, the value of *g* is negatively correlated with the support vectors (Dong et al. 2023). In addition, we evaluate the overall performance of the model by accuracy (Acc), F1‐score, and Matthews correlation coefficient (MCC). Among them, Acc is used to evaluate the classification correctness of the model, and the closer the value is to 1 indicates the better classification performance of the model. F1‐score provides a comprehensive evaluation of precision and recall, which is a kind of reconciled average of them, and the larger the value is, the better the balance between precision and recall. MCC is used as a measure of the strength of the linear relationship between the variables. The higher the value, the higher the correlation between the true and predicted values, and the better the prediction of the model. They are calculated as follows:

(3) $$\mathrm{Acc}=\frac{\mathrm{TP}+\mathrm{TN}}{\mathrm{TP}+\mathrm{FP}+\mathrm{TN}+\mathrm{FN}}$$

(4) $$\text{Precision}=\frac{\mathrm{TP}}{\mathrm{TP}+\mathrm{FP}}$$

(5) $$\text{Recall}=\frac{\mathrm{TP}}{\mathrm{TP}+\mathrm{FN}}$$

(6) $$F1-\text{score}=\frac{2\times \text{Precision}\times \text{Recall}}{\text{Precision}+\text{Recall}}$$

(7) $$\mathrm{MCC}=\frac{\mathrm{TP}\times \mathrm{TN}-\mathrm{FP}\times \mathrm{FN}}{\sqrt{\left(\mathrm{TP}+\mathrm{FP}\right)\left(\mathrm{TP}+\mathrm{FN}\right)\left(\mathrm{TN}+\mathrm{FP}\right)\left(\mathrm{TN}+\mathrm{FN}\right)}}$$

### 2.8 Statistical Analysis

The data were reported as mean ± standard deviation. Creating charts using Origin 2024 and Microsoft Excel 2021. Principal component analysis (PCA) and orthogonal partial least squares discriminant analysis (OPLS‐DA) were accomplished using Origin 2024 and the online analysis tool (https://cloud.metware.cn/). Finally, the operation process of the SVM model was all carried out in MATLAB 2023a.

## 3 Results and Discussion

### 3.1 Phenotype and Physiological State

Fruit phenotype is crucial in the identification of fruit shapes and quality and is a joint product of gene–environment interactions. Table S2 shows the phenotypic and physiological status data that we measured in this study. It can be seen that the phenotypic and physiological states of the different fruit shapes differed to varying degrees. There was no significant (*p* > 0.05) difference among the three fruit shapes in terms of width, with LFS being the longest and PCS the shortest in terms of length. Notably, the standard deviation of aspect ratio was much smaller than that of length and width. It suggests that aspect ratio is a more stable phenotype of LT population, which can be used as a characteristic index to distinguish each fruit shape. L*, a*, and b* represent the spatial coordinates of colors, and any color has a unique coordinate. Studies have reported that a* values are positively correlated with the degree of Maillard reaction (Li, Mo, et al. 2024; Li, Zhao, et al. 2024; Li, Zhu, et al. 2024). In the present study, the a* values from high to low were LFS > PCS > ES. It is possible that the differences in the abundance of browning substrates possessed by themselves resulted in different degrees of browning reaction during drying. Nevertheless, it was still difficult to distinguish the three fruit shapes by the naked eye (Figure S1). In addition, the b* values of PCS and LFS were significantly (*p* < 0.05) higher than those of ES, however, there was no significant difference in brightness (*p* > 0.05). There was no significant (*p* > 0.05) difference in soluble sugars among the three fruit shapes, while the difference in soluble proteins was highly significant. The soluble protein content in LFS (53.39 mg/g) was 1.43 times higher than that of ES (37.21 mg/g) and 1.85 times higher than that of PCS (28.91 mg/g). Soluble solids and titratable acids are closely related to flavor formation, and the ratio of soluble solids to titratable acids (solids‐to‐acid ratio, SA) is also often used in horticulture to evaluate fruit flavor and ripeness (Obenland et al. 2011). The order of soluble solids in different fruit shapes was PCS > ES > LFS, and the order of titratable acids was PCS > LFS > ES. However, the SA of the two compositions was highest for ES, followed by PCS and lowest for LFS. This indicates that there are some differences in flavor products between fruit shapes. Ash is an inorganic component that remains after undergoing a series of physical and chemical changes during the high temperature process, and PCS (79.39 mg/g) contained the highest amount of ash, LFS (72.55 mg/g) the second, and ES (66.39%) the lowest. Therefore, we hypothesized that PCS may be the richest in mineral elements. In addition, the total phenol content in ES (8.27 mg/g) was significantly higher than that in LFS (6.85 mg/g) and PCS (5.85 mg/g), probably because the antioxidant capacity of ES was higher than that of the other two fruit shapes. To further explore the association between LT phenotypes and physiological status, we further did a correlation analysis (Figure S3). The results showed that a* had a strong correlation with soluble solids and SA, with correlation coefficients of 0.90 and 0.94, respectively. Predicting that LT with a redder color might have a stronger aroma. Proteins and polyphenols form protein‐polyphenol complexes when heated or enzyme‐catalyzed thus causing changes in flavor and possessing greater antioxidant activity and stability (Yang et al. 2023). There was a strong negative correlation between soluble protein and ash (*r*, −0.92) and a strong positive correlation with total phenols (*r*, 0.99), as well as a strong negative correlation between total phenols and ash (*r*, −0.91). It may be that most of the proteins in LT during the drying process were combined with polyphenols to form a complex with covalent or noncovalent bonds, which promoted the formation of flavor substances and astringency in LT. And the alkali metals, alkaline earth metals and transition metals in the ash may play a catalytic role in the complex formation process, which resulted in a significant negative correlation between soluble sugars, total phenols and ash content. In addition, L* did not have strong correlations with other indicators, indicating that it is not desirable to judge the internal physiological state from the brightness of LT.

### 3.2 Broadly Targeted Metabolomics Analysis

#### 3.2.1 Metabolite Identification

**FIGURE 1:**
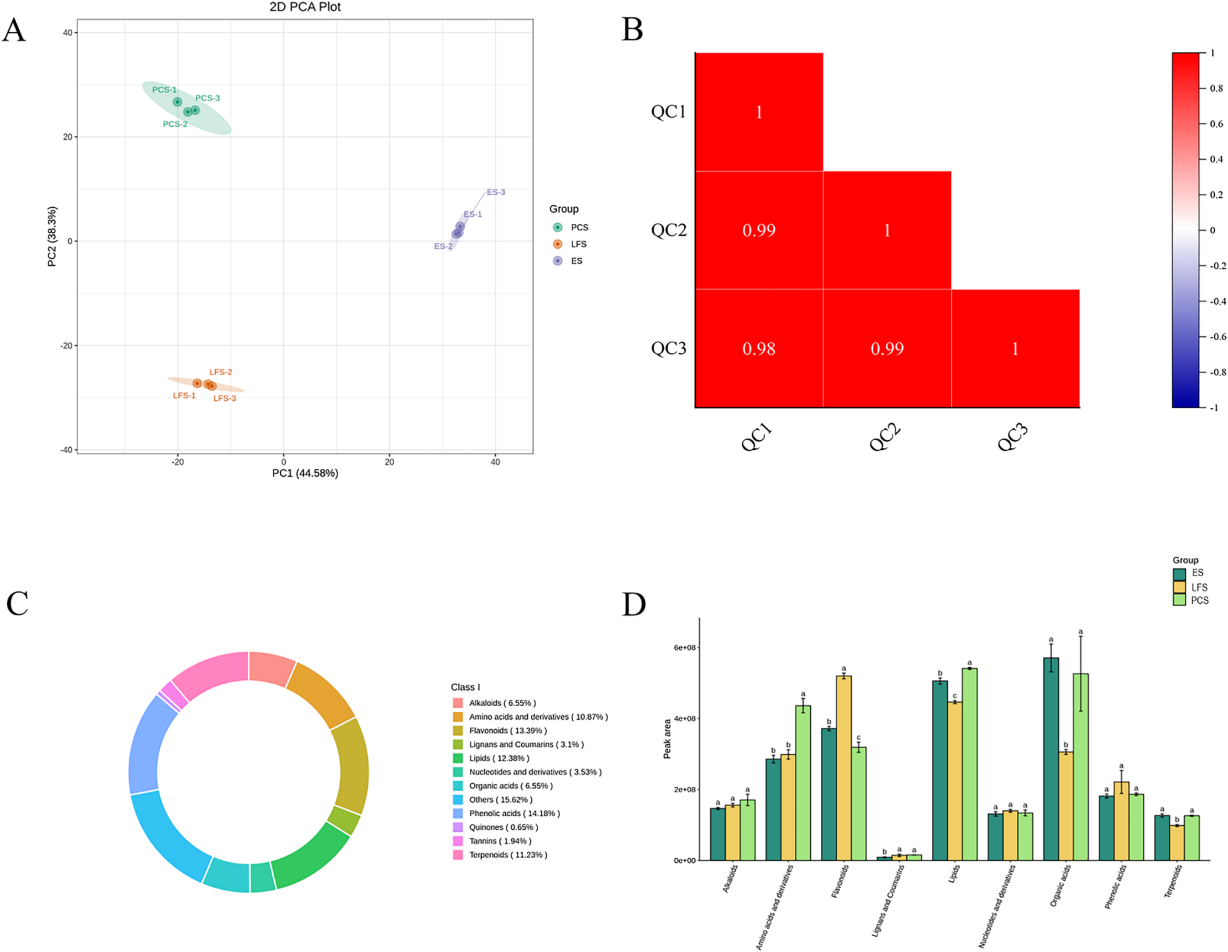
Identification of non‐volatile metabolites. (A) PCA scores of three fruit shapes. (B) Heat map of correlation between QC samples. (C) Primary classification and secondary classification of non‐volatile metabolites. (D) The relative content of non‐volatile substances, different letters mean a statistical difference (*p* < 0.05).

The researcher built a PCA model from the nonvolatile component data obtained by UPLC‐MS/MS for evaluating the metabolic level differences among the three fruit shapes. The results showed that PCS, ES, and LFS were significantly separated from each other with large spatial distances, indicating that the metabolic levels of the three fruit shapes differed significantly. In addition, PC1 and PC2 explained a total of 82.88% of the variance contribution, with small spatial distances between the samples in the same group, and the three QC samples were also clustered together with high correlation coefficients, which indicated that the experiments were reproducible, the methodology was reliable, and the data could be used for subsequent analyses (Figure 1A,B). Through a broad targeted metabolomics approach, a total of 1389 components were detected in the positive and negative ion modes (Appendix S2), which could be categorized into 12 types, with phenolic acids (197, 14.18%), flavonoids (186, 13.39%), lipids (172, 12.38%), terpenoids (156, 11.23%), and amino acids and their derivatives (151, 10.87%) were the major metabolites (Figure 1C). Phenolic acids and flavonoids are the major nonvolatile constituents of LT, and their biological activities such as antioxidant, hypoglycemic, and antidiabetic have been widely studied (Fan et al. 2023; Yang et al. 2022), and their contents are positively correlated with altitude and negatively correlated with longitude and latitude (Hu et al. 2024). Terpenoids are the predominant metabolites in LT and have biological activities such as antimicrobial, anti‐inflammatory, and anthelmintic (Pu 2022; Yang et al. 2022). In addition, terpenoids are also the most important constituents of volatile oil, and the pharmacopeia of the people's republic of China (2020 edition) states that the volatile oil content of LT should not be less than 1.4%. From the histogram, the content of organic acids, flavonoids, lipids, amino acids and their derivatives were higher. In addition, we found that the amount of lipids detected in the present study was second only to phenolic acids and flavonoids, and the total peak area of lipids in the three fruit shapes was higher than that of phenolic acids, suggesting that lipids have an important place in the metabolic profile of LT. The content of organic acids in ES and PCS was significantly (*p* < 0.05) higher than that in LFS, and flavonoids were mainly accumulated in LFS (Figure 1D). A previous study reported that LT phenolic acids and flavonoids were higher in fusiform than in ellipsoidal and round (Hu et al. 2024). However, in the present study, there was no significant difference in phenolic acids among the three fruit shapes (*p* > 0.05). This might be due to the differences in the detection methods and growth environments. The broad‐targeted metabolomics employed in this study differs from the former in terms of sensitivity and specificity. The selection of different chromatographic columns and mobile phase composition parameters may all affect the separation and quantitative results of phenolic acids. In addition, environmental factors such as light, temperature and humidity all affect the accumulation of phenolic acids. The fruit origin used in this study differs from that of the former. In the future, the sample size and the types of origin should be increased to further explore the relationship between phenolic acids and fruit shapes. However, the exploration of lipids by current researchers is much less than that of flavonoids and phenolic acids (Yang et al. 2022, 2023). Free fatty acids, diglycerides, and monoglycerides produced by enzymatic hydrolysis of lipids can be further oxidized into volatile substances such as alcohols, aldehydes, ketones, and esters, which are one of the important biochemical reactions for the production of flavor substances (Wang, Gao, et al. 2024; Wang, Wang, et al. 2024; Wang, Xie, et al. 2024). The flavor of LT is produced with the drying process, but the mechanism and substrate of flavor substance production in LT are not clear, and there are few related researches, and lipids may be a good perspective.

#### 3.2.2 Differential Metabolite Screening

**FIGURE 2:**
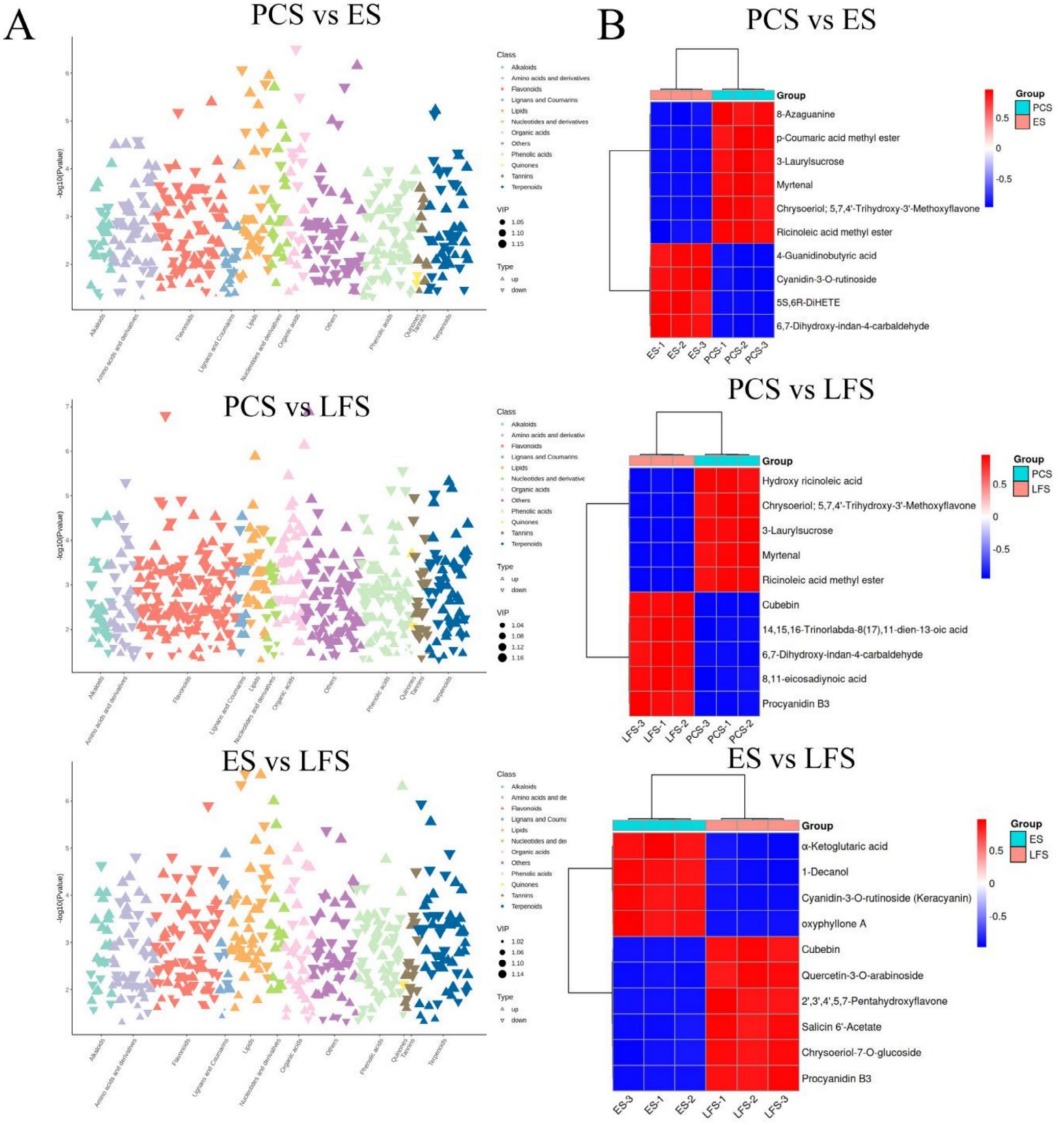
Difference of nonvolatile metabolites and analysis of antioxidant activity. (A) The differential metabolite classification scatter plots of PCS vs. ES, PCS vs. LFS, and ES vs. LFS, respectively. (B) Clustering heat maps of VIP top 10 differential metabolites in each comparison group.

OPLS‐DA is an effective method to screen for differential metabolites, and the obtained variable importance in projection (VIP) values can be used for further analysis (Huang 2024). The score plots showed that PCS, ES, and LFS were significantly separated from each other, and the variance explained by PC1 and PC2 was in the range of 78.77%–79.73% (Figure S4A), and the results of 200 permutation test indicated that the model was not overfitted (Figure S4B). To further screen for differential metabolites across fruit shapes, we screened for differential metabolites among PCS, ES, and LFS using *p* < 0.05, VIP > 1, and |log_2_FoldChange| > 1 as thresholds. The results showed that PCSvsES had 420 differential metabolites (up‐regulated 253, down‐regulated 167), and the metabolite type was dominated by flavonoids (17.6%). ESvsLFS had 522 differential metabolites (up‐regulated 282, down‐regulated 240), which were also dominated by flavonoids (26.4%). PCSvsLFS had 438 differential metabolites (up‐regulated 261, down‐regulated 177), dominated by flavonoids (17.1%), terpenoids (13.2%), and phenolic acids (12.5%), while 64% of flavonoids were downregulated (Figure 2A). It can be seen that flavonoids, phenolic acids, and terpenoids were the main sources of differences among the three fruit shapes. We used the top 10 differential metabolites of VIP as biomarkers of fruit shape differences (Figure 2B). Glycosides in LT such as kaempferol‐3‐*O*‐rutinoside and naringenin have the potential ability to inhibit α‐glucosidase activity (Wang et al. 2015). In addition, quercetin‐3‐*O*‐arabinoside is a group sensing inhibitor, myrtenal has an aromatic peachy and woody flavor and is one of the important components in LT essential oils, but it is only present in PCS, which may affect the overall flavor of different fruit shapes. 1‐decanol, on the other hand, is a straight‐chain fatty alcohol that also has a sweet, floral, and fruity aroma, and is an important component of chemical and flavor plants, often produced from natural sources through the hydrogenation of related fatty acids in coconut oils and waxes. However, LT has a significant amount of 1‐decanol, especially ES. Therefore, LT can be used as a potential source of 1‐decanol feedstock, thus replacing non‐renewable oil consumption and deforestation (Chen and Gonzalez 2023). Procyanidin B3 contributes to the purplish‐red color in the sample, and has and good antioxidant and anti‐inflammatory properties, which neutralize free radicals and counteract cellular oxidative stress (Zhu et al. 2023). This may also contribute to the significantly higher a* in LFS than in ES and PCS. It is noteworthy that myrtenal, and 3‐laurylsucrose are only present in PCS, and cubebin, 2′,3′,4′,5,7‐pentahydroxyflavone are only present in LFS, which can be used as exclusive biomarkers for their respective fruit shapes.

In addition, there were 100 common differential metabolites between the three comparison groups. These included 28 flavonoids, 16 terpenoids, 14 phenolic acids, 6 lipids, 6 tannins, 5 amino acids and derivatives, 4 nucleotides and their derivatives, 4 alkaloids, 4 organic acids, 2 lignocoumarins, 1 quinone, and 10 others (Figure S5A, Table S3). Based on the expression amount of each metabolite, we performed a cluster analysis of the 100 differential metabolites using the K‐means clustering algorithm. Based on the clustered cluster elbow diagram, we divided all the metabolites into four clusters (Figure S5B), which clustered 29, 24, 13, and 34 metabolites (Figure S5C), respectively. The metabolites in Cluster 1 and Cluster 2 had higher accumulation in PCS and ES, respectively, and both Cluster 3 and Cluster 4 had higher accumulation in LFS. Notably, Cluster 3 and Cluster 4 species were dominated by terpenoids (8, 61.54%) and flavonoids (14, 41.18%), respectively. To further analyze the intrinsic reasons for the differences in metabolic levels among the three fruit shapes, we performed Kyoto encyclopedia of genes and genomes (KEGG) pathway analysis on the 100 common differential metabolites screened. The results showed that the differential metabolites responded significantly (*p* < 0.05) in the flavone and flavonol biosynthesis, pentose and glucuronate interconversions pathway (Figure S5D).

#### 3.2.3 Antioxidant Activity

Due to the different detection methods, it is difficult to evaluate the antioxidant capacity of the samples in a single experiment, so we chose DPPH, ABTS, and FRAP to comprehensively evaluate the antioxidant and free radical effects of different fruit shapes. The results showed that all three fruit shapes of LT had significant antioxidant activities, but differed in intensity (Table S4). First, ES (130.34 μmol Fe^2+^/g) had the strongest electron transfer capacity for Fe^2+^, which was significantly higher than that of LFS (58.00 μmol Fe^2+^/g) and PCS (43.68 μmol Fe^2+^/g). It suggests that ES may have the strongest free radical scavenging ability, which was verified in the DPPH and ABTS scavenging experiments. The scavenging rates of ABTS and DPPH were both significantly (*p* < 0.05) higher in ES (90.24%, 61.93%) than in LFS (77.93%, 34.14%) and PCS (63.54%, 25.61%). All these results may be attributed to the abundant total phenolic content in ES. According to research reports, secondary metabolites such as kaempferol‐3‐*O*‐rutinoside, naringin, hannogenol, catechin, and protocatechin in LT have significant antioxidant activity (Fan et al. 2023; Yang et al. 2022), and in order to screen for potential antioxidant actives in LT, we correlated the differential metabolites with antioxidant capacity of the various fruit shapes in a Mantel test correlation analysis. The results showed that nine differential metabolites showed a strong positive correlation (*r* > 0.8, *p* < 0.01) with antioxidant activity (Figure S5E), including two flavonoids (2′‐hydroxy‐2‐methoxychalcone, 1,3,6,8‐tetrahydroxy‐2,5‐dimethoxyxanthen‐9‐one), two sugars (3′‐palmitoyl sucrose, 3′‐linoleoyl sucrose), 1 phenolic acid (3,4‐dihydroxyphenethyl alcohol‐8‐*O*‐[4‐*O*‐caffeoyl‐β‐d‐apinosyl(1 → 3)‐β‐d‐glucosyl(1 → 6)]‐β‐d‐glucoside), 1 triterpene (2,3‐dihydroxyoleana −11,13(18)‐dien‐28‐oic acid), a tannin (2α,3α‐epoxy‐5,7,3′,4′‐tetrahydroxyflavan‐(4β → 8)‐epicatechin), a ketone (2,5‐dihydroxyacetophenone) and an organic acid (2,6‐diaminooimelic acid). Among them, 3′‐linoleoyl sucrose and 1,3,6,8‐tetrahydroxy‐2,5‐dimethoxyxanthen‐9‐one showed strong positive correlations with FRAP, ABTS, and DPPH. 2,5‐dihydroxyacetophenone is a natural product isolated from *Radix Rehmanniae*. It is a natural product with anti‐inflammatory, anxiolytic, and neuroprotective effects. 2′‐hydroxy‐2‐methoxychalcone is an important intermediate of flavonoids, which not only has significant antioxidant activity, but also inhibits lipoxygenase activity. 2′‐dihydroxyacetophenone is an important intermediate of flavonoids, which not only has significant antioxidant activity, but also inhibits lipoxygenase activity. In addition, 2,6‐diaminooimelic acid forms stable cell wall components, and bacteria such as escherichia coli produce lysine through the action of diaminoheptanedioic acid decarboxylase (Liu et al. 2023).

### 3.3 Aroma Quality Analysis Based on E‐Nose

**FIGURE 3:**
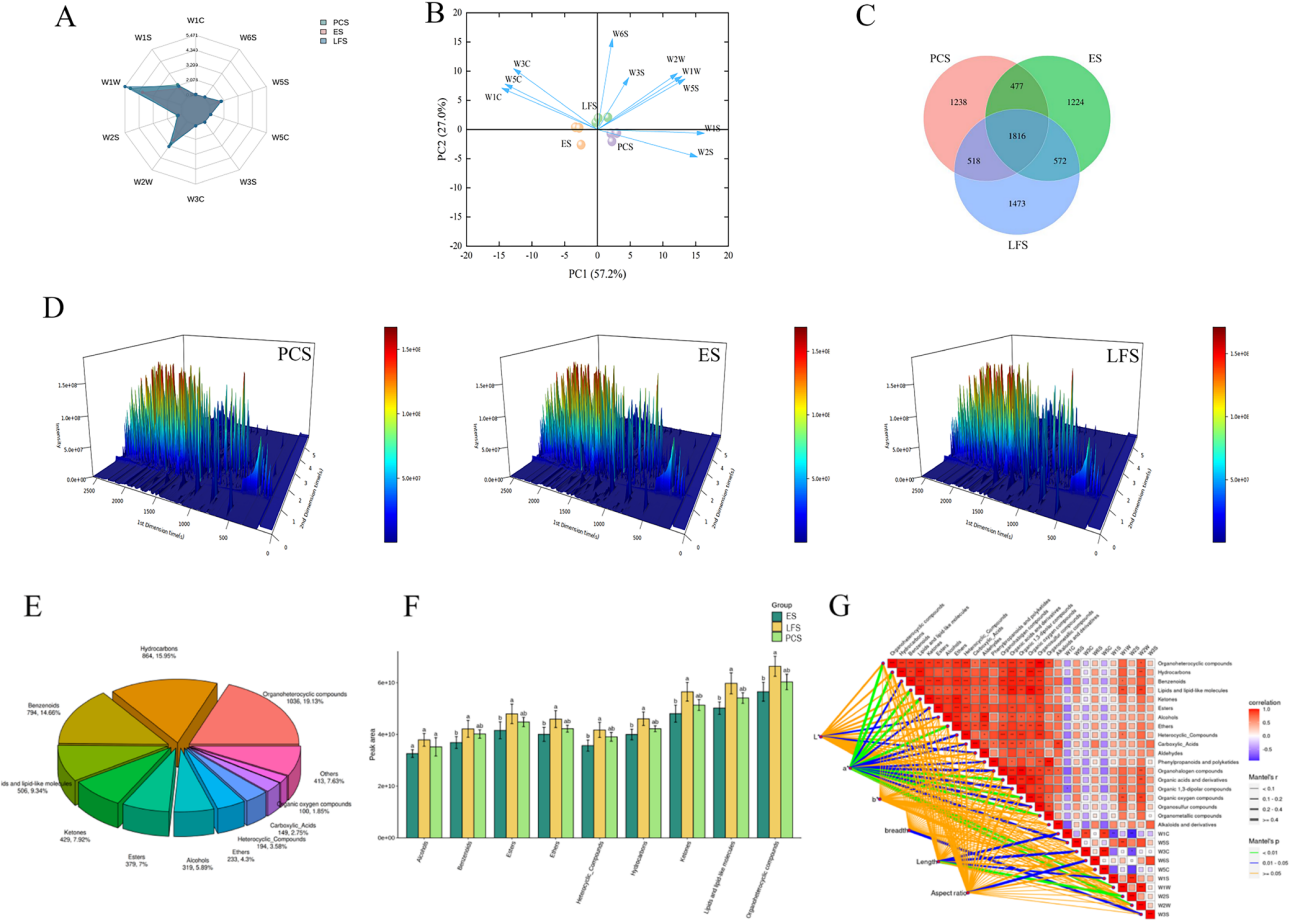
Identification of volatile substances. (A) Radar plots of the response strength of the 10 sensors in the electronic nose in the three fruit shapes. (B) PCA double labeled chart. (C) Wayne's chart for volatile substances. (D) Three‐dimensional total ion flow chromatogram. The horizontal coordinate is the one‐dimensional retention time (s) and the vertical coordinate is the two‐dimensional retention time (s). The color and peak height tell the intensity of the ion response. The redder the color, the higher the response intensity. (E) Pie chart of volatile substances classification. (F) The relative content of volatile substances, different letters mean a statistical difference (*p* < 0.05). (G) Manteltest analysis of phenotypes and volatiles.

In order to clarify the difference in aroma quality among the three fruit shapes, the average values of the data sensors were calculated for samples of the same fruit shape at the point of performance stabilization time (147th, 148th, and 149th s). Figure 3A shows the response intensities of the 10 sensors through radar plots, which are closely related to the content of the compounds. It can be observed that there are differences in the response intensities of the 10 sensors. This indicates that the three fruit shapes have different contents of volatiles. Notably, all three fruit shapes had high responses in the WIW and W2W sensors, indicating that the aroma components in LT are mainly contributed by sulfides, aromatic components, and organ sulfides. In addition, all three fruit shapes also showed significant differences in W1W, indicating that the differences in their sulfide contents were large. The double‐label plot showed that PC1 and PC2 provided a high variance explained (84.2%), and the three fruit shapes were significantly separated, indicating that the overall flavor of the three fruit shapes was significantly different (Figure 3B). In addition, W1S and W2S were closer to PCS, W6S and W3S were closer to LFS, and W1C, W3C, and W5C were closer to ES, and the three sensors were in the negative semiaxis of the PC1 direction, indicating that the aromatic components, the short‐chain aromatic components, and the nitrogen‐based components contributed more to the flavor of ES. W1S and W2S were close to PCS in the fourth quadrant, indicating that the methane and ethanol made a significant contribution to the flavor of PCS. Similarly, the flavor substances in LFS were mainly contributed by sulfides, aromatic components, small molecule nitrogen oxides, alkanes, and hydrides.

### 3.4 Flavoromics Analysis

#### 3.4.1 Identification of Flavor Substances

The three‐dimensional total ion flow chromatograms showed an abundance of volatiles in LT, and numerous metabolites had high ion response intensities (Figure 3D). The PubChem database and ClassyFire software were used to analyze the species annotation of the detected flavor substances and to analyze the number and relative content of flavor substances corresponding to each class (Djoumbou Feunang et al. 2016). By annotating and decontaminating the detected substances, 4049, 4089, and 4379 flavor substances were identified in PCS, ES, and LFS, respectively, with 1816 common flavor substances (Figure 3C). A total of 7321 flavor substances were detected in the three fruit shapes, of which 1906 could not be accurately annotated to a specific class, and the remaining 5413 flavor substances were classified into 26 classes, organo heterocyclic compounds (1036, 19.40%), hydrocarbons (864, 16.18%), benzenoids (794, 14.87%), lipids and lipid‐like molecules (506, 9.47%), ketones (429, 8.03%), and esters (397, 7.10%) were the major components (Figure 3E). In the early exploration of flavor substances in LT, only a few volatiles such as 1,8‐cineole and (*E*)‐2‐decenal could be extracted due to technical limitations (Liang et al. 2023). To date, more than 600 volatile substances have been detected in LT and attempts have been made to extract them by steam distillation, headspace solid‐phase microextraction, microwave hydrodistillation extraction, etc. (Liang et al. 2024). In terms of content, LFS consistently contained more volatile components than ES and PCS (Figure 3F). This is similar to the results of a* and electronic nose, so we speculate that there may be some correlation between the color and aroma of LT. Combined with the folklore that LT aroma is correlated with length, therefore, we LT phenotypes were analyzed with volatiles and e‐nose sensors for Manteltest. The results showed that a* had a significant (*p* < 0.05) positive correlation with W1W and W2W (*r*, 0.69; *r*, 0.77), whereas b* and L* did not have significant correlations (*p* > 0.05) with either volatile matter or electronic nose sensor. In addition, length also had a significant (*p* < 0.05) positive correlation with WIC (*r*, 0.61). This suggests that the redder the color of LT, the more aromatic components and sulfides it may contain, while longer LT may have more aromatic substances.

#### 3.4.2 Differential Metabolite Screening

**FIGURE 4:**
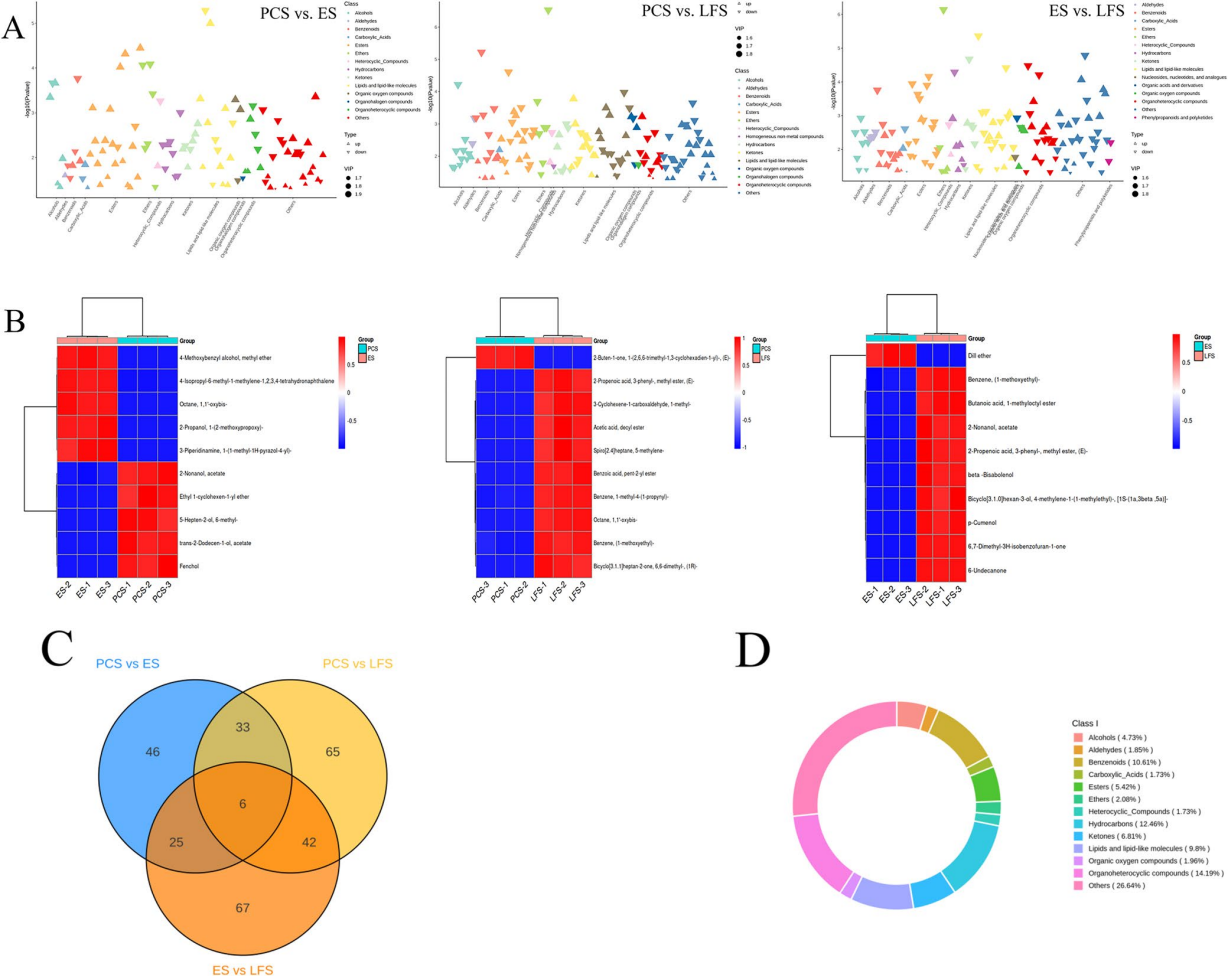
Analysis of differences in volatile substances. (A) the differential metabolite classification scatter plots of PCS and ES, PCS and LFS, ES and LFS, respectively. (B) clustering heat maps of VIP top 10 differential metabolites in each comparison group. (C) Wayne chart. (D) Differential metabolite substance classification.

As with the broadly targeted metabolome, we screened for the presence of differential metabolites between LFS, ES, and PCS at *p* < 0.05, VIP > 1, and |log_2_FoldChange| > 1. The results showed that there were 100 differential metabolites in PCSvsES, dominated by esters (19.0%) and heavily up‐regulated in PCS (90.4%); there were 146 differential metabolites in PCSvsLFS, dominated by esters (13.6%) and Lipids and lipid‐like molecules (13.0%) dominated; 140 differential metabolites in ES versus LFS, dominated by lipids and lipid‐like molecules (15.0%), esters (12.1%), and organoheterocyclic compounds (12.1%), and 94.1% of esters were down‐regulated (Figure 4A). Thus, esters were the main metabolite type that differed among the three fruit shapes and accumulated the least in ES. One of the main sources of aroma in LT fruits, the drying process leads to the accumulation of ester components and thus enhances the aroma (You et al. 2024). We screened the 10 with the largest VIP values as biomarkers (Figure 4B). The heatmap showed that nine flavor substances were up‐regulated in ES and PCS in LFS, which again indicated the high flavor content in LFS, and again verified the folklore that length is positively correlated with aroma. Wayne plots revealed six common differential flavor substances among the three fruit shapes (Figure 4C). The volatile components detected were much higher than the nonvolatile components, but the number of differential metabolites screened was significantly lower, probably because the differences in the types and contents of volatiles among the three fruit shapes were smaller than those of nonvolatiles. Due to the demand for subsequent analysis, we screened 868 differential metabolites between PCS, ES, and LFS again at *p* < 0.05, VIP > 1, including 123 organoheterocyclic compounds, 108 hydrocarbons, 92 benzenoids, 85 lipids and lipid‐like molecules, 59 ketones, 47 esters, 41 alcohols, 18 ethers, 17 organic oxygen compounds, 16 aldehydes, 15 carboxylic_acids, 15 heterocyclic_compounds, and 231 others (Figure 4D).

#### 3.4.3 Aroma Characterization and Relative Odor Activity Method (ROAV)

**FIGURE 5:**
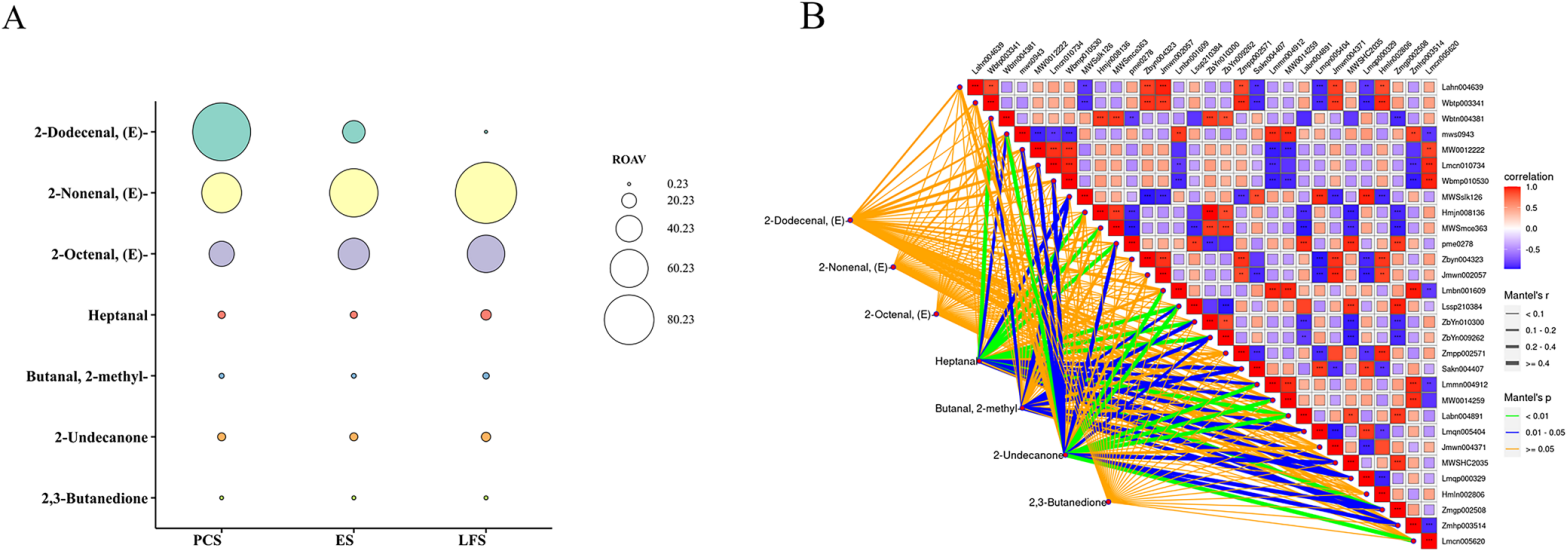
Flavor characterization of volatiles. (A) ROAV bubble diagram of key flavor substances; (B) Manteltest analysis of non‐volatile and volatile substances (demonstration of the top 30 non‐volatile substances of high relevance).

We screened five aldehydes (2‐dodecenal, (*E*)‐2‐nonenal, (*E*)‐2‐octenal, (*E*)‐, heptanal, butanal, 2‐methyl‐) and two ketones (2‐undecanone, 2,3‐butanedione) as key flavor substances in LT. It indicates that aldehydes and ketones are the main flavor substances in LT, which is consistent with previous studies (Liang et al. 2024, 2025). It is worth noting that 2‐undecanone and 2‐dodecenal were first identified as the key aroma sources in LT. This might be because the high sensitivity of the GC × GC‐TOFMS technology detected some substances with low concentrations but significant aroma in LT. aldehydes are the main contributors of flavor substances in many spices due to their low olfactory threshold and unique odor characteristics. The ROAV values of 2‐nonenal, (*E*)‐2‐octenal, (*E*)‐2‐dodecenal, (*E*)‐ were significantly higher than those of the other flavor substances (except for 2‐dodecenal, (*E*)‐ in the LFS) as can be seen from the bubble diagrams, and their contribution to the flavor of the LT was significant (Figure 5A, Table 1). They were the main contributors to the green, fatty, sweet, cucumber, and nuts odors in LT. Although the two ketones, 2‐undecanone and 2,3‐butanedione, had lower ROAV values than the other five aldehydes, they provided the significant orange, fresh, green, pleasant, buttery aroma profile found in LT (Fu et al. 2026; Hu et al. 2025).

**TABLE 1:** Seven flavor substances with ROAV values greater than 1.

Flavor substance	Class	1st Dimension time (s)	2nd Dimension time (s)	CAS	Formula	Range of odor	Odor character	PCS_ROAV	ES_ROAV	LFS_ROAV
2‐Dodecenal, (*E*)‐	Aldehydes	1736.89	2.018333333	20407‐84‐5	C12H22O	0.00053	Green, Fatty, Sweet	94.57	33.58	0.23
2‐Nonenal, (*E*)‐	Aldehydes	1289.92	1.834888889	18829‐56‐6	C9H16O	0.0002	Fatty, Cucumber	63.47	77.95	100.00
2‐Octenal, (*E*)‐	Aldehydes	1124.596667	1.788444444	2548‐87‐0	C8H14O	0.003	Nuts, Green, Fatty	38.16	48.77	59.33
Heptanal	Aldehydes	713.954	1.887333333	111‐71‐7	C7H14O	0.003	Citrus, Fatty, Rancid	7.54	7.23	12.80
Butanal, 2‐methyl‐	Aldehydes	317.98	1.614333333	96‐17‐3	C5H10O	0.001	Cocoa, Almond	3.77	3.25	5.97
2‐Undecanone	Ketones	1379.91	2.118666667	112‐12‐9	C11H22O	0.004355	Orange, Fresh, Green	8.76	8.74	10.83
2,3‐Butanedione	Ketones	392.5464286	1.521857143	431‐03‐8	C4H6O2	0.002	pleasant, buttery	1.62	1.92	1.91

*Note:* 1st Dimension time (s): one‐dimensional retention time (in seconds); 2nd dimension time (s): 2D retention time (in seconds); range of odor: concentration values for flavor characteristics.

However, the ROAV values still differed among the three fruit shapes. 2‐nonenal, (*E*)‐ ranked LFS (100.00) > ES (77.95) > PCS (63.48) among the three fruit shapes, 2‐dodecenal, (*E*)‐ ranked PCS (94.57) > ES (33.59) > LFS (0.23), 2‐octenal, (*E*)‐ was ranked LFS (59.33) > ES (48.77) > PCS (38.16). Notably, 2‐dodecenal, (E)‐ showed the most significant difference among the three fruit shapes, with ROAV values in LFS being 2.82 and 411.17 times higher than those in ES and PCS, respectively. (*E*)‐2‐octenal, (*E*)‐2‐dodecenal have been previously reported and shown by recombination and omission experiments to be significant (*p* < 0.05) contributors to flavor in LT (Liang et al. 2024). They may be produced by autoxidation or enzymatic oxidation of unsaturated fatty acids under the influence of catalase (Liang et al. 2025). Two other aldehydes, heptanal and butanal, 2‐methyl‐ contributed to the odor of citrus, fatty, rancid, cocoa, and almond. Although the two ketones, 2‐undecanone and 2,3‐butanedione, had lower ROAV values than the other five aldehydes, they provided the significant orange, fresh, green, pleasant, buttery aroma profile found in LT. The aroma of ketones is influenced by the molecular structure; generally, low carbon fatty ketones have weak aroma, and C7‐C12 asymmetric ketones have strong aroma (Xu et al. 2019), which may be the reason for the higher ROAV value of 2‐undecanone than that of 2,3‐butanedione in LT.

### 3.5 Combined Analysis of Non‐Volatile and Flavor Substances

We analyzed 100 differential nonvolatile metabolites with 7 flavor substances with ROAV > 1. The results showed that four flavor substances, 2‐dodecenal, (*E*)‐, 2‐undecanone, butanal, 2‐methyl‐, and heptanal, had 119 pairs of significant correlations (*p* < 0.05) with nonvolatile substances (Figure 5B, Appendix S3). Among them, 82 had strong positive correlations (*r* > 0.67), including 19 flavonoids, 12 phenolic acids, 9 lipids, 7 terpenoids, 6 nucleotides and derivatives, 6 organic acids, 5 alkaloids, 2 amino acids and derivatives, 2 tannins, and 14 others. 37 were strongly negatively correlated (*r* < 0.67), including 11 flavonoids, 10 terpenoids, 3 phenolic acids, 2 lipids, 1 nucleotide and derivatives, 1 alkaloid, 1 amino acid and derivatives, 1 quinone, 1 tannin, and 4 others. Therefore, flavonoids and phenolic acids in LT are most closely related to flavor substances. This may be due to the enzymatic hydrolysis of the flavonoid glycosides of LT during the drying process, and the flavor components captured in the glycosides are released during the production of flavonoids, enhancing the flavor of LT (Wang, Gao, et al. 2024; Wang, Wang, et al. 2024; Wang, Xie, et al. 2024).

### 3.6 Chemical Information Fingerprinting by FT‐NIRS

**FIGURE 6:**
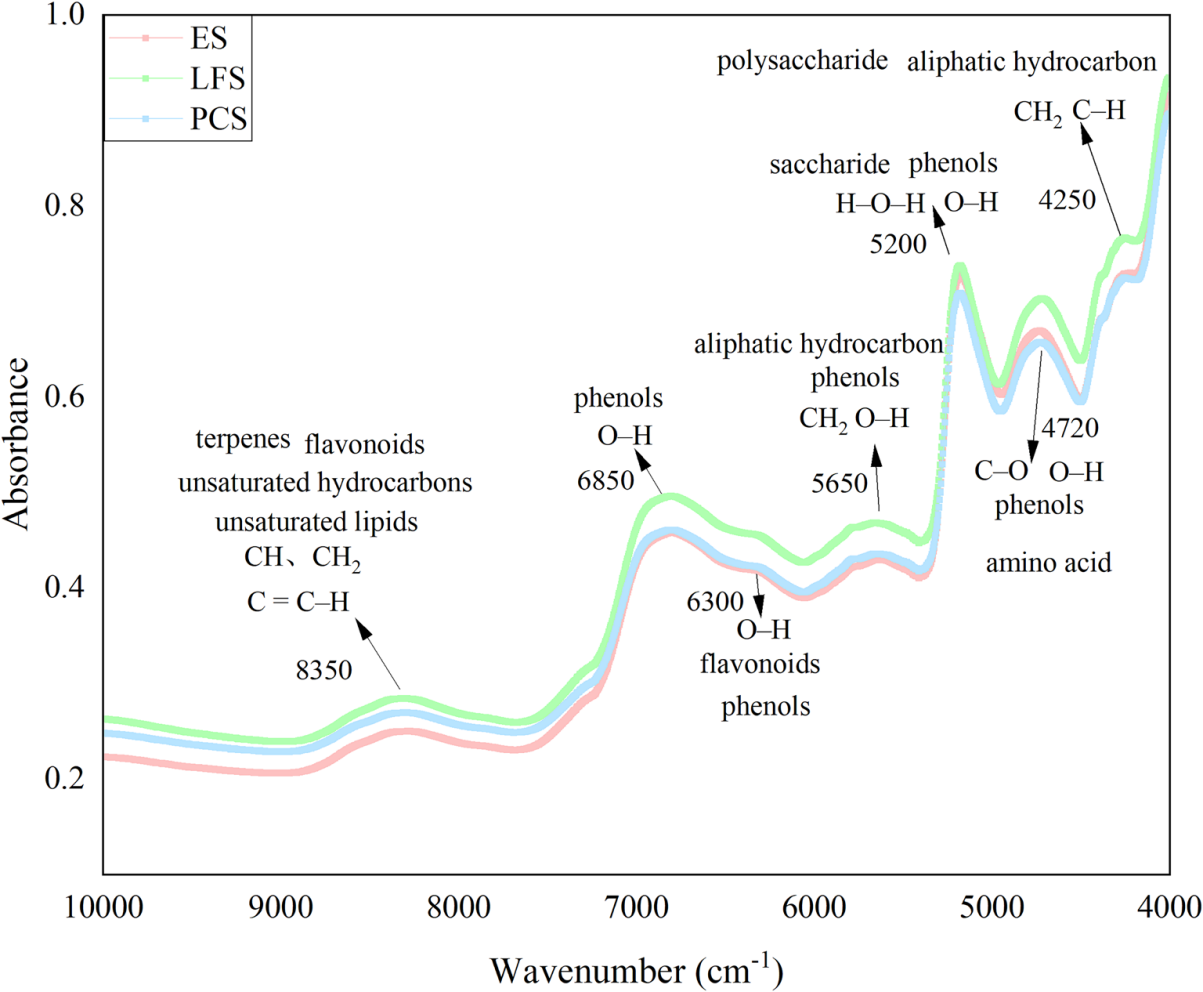
Average spectrogram of the three fruit shapes.

As shown in Figure 6, LT has seven common characteristic peaks in the range of 10,000–4000 cm^−1^ for different fruit shapes, with similar peak shapes and peak positions, and the differences are more reflected in the absorbance. There is a strong absorption band near 8350 cm^−1^, which is related to the 2nd overtone of C=C—H stretching, the 2nd overtone of the CH bond formed by the CH_2_ and CH groups and C‐H stretching first and second overtones, which may be caused by unsaturated lipids, unsaturated hydrocarbons, terpenes, or flavonoids (Elrasheid Tahir et al. 2023; Liu et al. 2019). The absorption band near 6850 cm^−1^ corresponds to the first overtone of O—H stretching resp. may be related to phenolic compounds (Liu et al. 2022). 6300 cm^−1^ may be related to O—H stretching and combination bands associated with phenolics, flavonoids. The absorption band near 5650 cm^−1^ corresponds to symmetric CH_2_ binding vibrations, the first overtone of O—H stretching, which may be related to phenolic and aliphatic compounds (Drees et al. 2023). 5200 cm^−1^ may be related to phenolic compounds (Liu et al. 2022). The absorption band near 5200 cm^−1^ may be related to O‐H stretching and H—O—H bending caused by sugars or phenols (Chen et al. 2023). The strong absorption band near 4720 cm^−1^ may have the first overtone of C—O—H stretching and H—O—H bending in phenols and amino acids. The first overtone of C—O with amide III, the O—H first overtone, and O—H stretching vibration are related (He, Shi, et al. 2024; He, Yang, and Wang 2024). The absorption band near 4250 cm^−1^ corresponds to the second overtones of C—H and CH_2_ bending, the first overtone of —CH_2_ symmetric stretching and —CH_2_ bending modes, which may be related to aliphatic hydrocarbons, polysaccharide compounds (Deng et al. 2024). Overall, the absorption peaks of the three fruit shapes are roughly the same, but there are some subtle differences.

### 3.7 SVM Model

The above results showed that the three fruit shapes differed significantly in phenotype, physiology, antioxidant capacity, and metabolite composition, suggesting that they differ in their use value. However, the identification of different fruit shapes of LT in the market often requires extensive experience and expertise, which is extremely unfriendly to ordinary consumers. Therefore, a rapid identification model of the three fruit shapes is necessary. Before modeling, we divided the test set and training set by Kennard‐Stone (K‐S) algorithm in the ratio of 7:3. The modeling results are shown in Table 2. Compared with the original data, preprocessing can significantly improve the classification performance of the model. Among them, the 2nd preprocessing has the best effect; the correct rate of classification reaches 1, and the MCC value and F1 value both reach 1. SVM is able to classify linearly in high dimensions, but with the increase of the number and type of samples, SVM is easy to overfit. Therefore, it is necessary to increase the fruit shape of LT and the sample size to evaluate the robustness of the model in the future.

**TABLE 2:** SVM model parameters based on different preprocessing methods.

Pretreatment	*c*	*g*	Train set			Test set		
			Acc	F1‐score	MCC	Acc	F1‐score	MCC
None	5.7296	24.9539	0.7143	0.6722	0.4966	0.6667	0.7407	0.5727
MSC	0.1650	12.5799	0.7619	0.7682	0.6415	0.8889	0.9026	0.8488
SNV	0.4120	12.2767	0.7619	0.7682	0.6415	0.8889	0.9026	0.8488
MSC + SNV	0.4232	14.8173	0.7619	0.7682	0.6415	0.8889	0.9026	0.8488
1st	1.7516	2.2824	0.7143	0.7746	0.6316	0.7800	0.8198	0.7077
2nd	0.7744	2.8944	0.8571	0.8780	0.8092	1.0000	1.0000	1.0000
Row	5.7296	24.9539	0.7143	0.6722	0.4966	0.6667	0.7407	0.5727

### 3.8 Correlation Analysis of FT‐NIRS With Metabolites

Metabolomics and FT‐NIRS have been shown to respond to the rich chemical information in different fruit shapes of LT, but the correlation of spectral bands with specific components in the samples has not been explored. This gap can limit the interpretability of the FT‐NIRS data in terms of the underlying chemical changes. In this regard, hyperspectral datasets have been correlated with elements and metabolites to enhance the interpretability of the dataset (He, Shi, et al. 2024; He, Yang, and Wang 2024; Sun et al. 2019). In addition, although broadly targeted metabolomics and GC × GC TOF MS provide rich chemical information, they also face some challenges such as high cost and complex sample preparation processes. These factors may hinder the realization of efficient, nondestructive, high‐throughput analysis. To address these limitations, it is crucial to develop strategies that combine the advantages of both datasets while minimizing the disadvantages. This study provides a more comprehensive and efficient method for the identification and rapid detection of fruit shapes of LT. We correlated the nonvolatiles and volatiles with the spectral bands by pearson correlation analysis to screen out the bands that were strongly correlated with the metabolites. The results showed that 5‐*O*‐caffeoylshikimic acid, quercetin‐3‐*O*‐α‐l‐arabinofuranoside, quercetin‐3‐*O*‐galactoside, quercetin‐3‐*O*‐glucoside, and quercetin‐5‐*O*‐β‐d‐glucoside five nonvolatiles had 1431 strong positive correlations (*r* > 0.8) with FT‐NIRS; the three volatiles, 2‐nonenal, (*E*)‐, butanal, 2‐methyl‐, and heptanal had 1606 positive correlations (*r* > 0.5) with FT‐NIRS; and the three sensors, W1W, W2W, and W5S, had 1702 strong positive correlations (*r* > 0.8) with FT‐NIRS (Appendix S3). In addition, after a literature review, the telescopic vibrations of chemical bonds and groups of nonvolatiles and volatiles appeared in their respective strongly correlated bands (Table S5). Therefore, we hypothesized that the chemical bonds and groups in these metabolites in LT were dispersed in these bands and the intensity of absorbance could reflect the content of these metabolites to some extent. It is noteworthy that the telescopic vibrations of chemical bonds and groups associated with flavor substances and sensors are concentrated at the ends of the full band, whereas the telescopic vibrations of chemical bonds and group sets of nonvolatile substances are in the middle band. Overall, the correlation of metabolites with the spectral dataset effectively explains the link between spectral bands and chemical composition. This correlation not only enhances the interpretability of the spectral features, but also emphasizes that FT‐NIRS can be used as a non‐destructive, efficient, and cost‐effective means of chemical composition detection in different fruit shapes of LT.

### 3.9 Limitations and Future Prospects

The fruit type categories selected in this study are not diverse enough to represent the diverse phenotypes of LT in complex environments. In the future, it is necessary to expand the sample sources to cover more fruit type categories under different ecological environments. At the same time, increase the sample quantity to improve the reliability of the samples and more effectively explore the metabolic pathway mechanisms of fruit type differences. Moreover, we are only limited to the phenotypes and secondary metabolic differences of the fruits, ignoring factors such as the growth stage and maturity of fruit development. Later, more time points during the growth process of LT should be considered to deeply reveal the mechanism of fruit expansion during the growth process. Finally, it is necessary to incorporate transcriptomics and genomics methods to reveal the fundamental causes of fruit type differences at the genetic level. This is the necessary path to achieve precise regulation of the quality of LT.

## 4 Conclusion

In this study, we comprehensively characterized the three fruit shapes of LT by integrating phenotype, physiology, chemical composition and FT‐NIRS. The results showed that there were some differences and associations between the color and physiological status of the different fruit shapes. More soluble proteins were accumulated in LFS, the highest total phenol content was found in ES, and titratable acid was mainly found in PCS. In addition, the differential metabolites of the three fruit shapes also responded significantly in the pathway of lavone and flavonol biosynthesis. The response of flavonoids and their associated pathways may be due to the survival environment. Antioxidant experiments showed that 3′‐linoleoyl sucrose, 1,3,6,8‐tetrahydroxy‐2,5‐dimethoxyxanthen‐9‐one had a strong positive correlation with all three antioxidants, and were the main active substances in LT acting as antioxidants. The electronic nose results showed that the main aroma components in LT were aromatics and sulfides and differed among the three fruit shapes. The flavoromics approach of GC × GC‐TOFMS was applied for the first time to LT and demonstrated a strong potential, with several times as many metabolites being detected as before. 2‐nonenal, (*E*)‐2‐octenal, (*E*)‐2‐octenal, (*E*)‐, contributed the most to the aroma of LT, with the first two mainly accumulated in the LFS. In addition, correlation analysis showed that flavonoids and phenolic acids were closely related to the formation of LT flavor. Finally, we hypothesized the possible bands of chemical bonds and groups associated with five nonvolatiles, three volatiles, and three electronic nose sensors by correlation analysis of the FT‐NIRS dataset with metabolites. In our opinion, the significance of this study lies in revealing the differences and intrinsic connections among the phenotypes, physiology and chemical substances of different fruit shapes of LT, providing directions for the formation of the aroma of LT, and enriching the quality assessment system of LT.

## Author Contributions

**Tianmei Yang:** data curation, investigation, resources. **Jinyu Zhang:** writing – review and editing, funding acquisition, project administration. **Yuanzhong Wang:** investigation, methodology, writing – review and editing. **Meiquan Yang:** investigation, conceptualization. **Dengke Fu:** investigation, writing – original draft, formal analysis, methodology, software, conceptualization. **Weize Yang:** data curation, project administration, visualization, resources. **Zongliang Xu:** supervision, validation, resources.

## Funding

This work was supported by Support Program for Talents of Developing Yunnan, XDYC‐CYCX‐2022‐0027, Yunnan Province Major Project on Biomedical Sciences: Research on Innovation in *Lanxangia tsaoko* Seed Industry, 202502AS100009‐03, Specialty Team for the Chinese Herbal Medicine Industry of Yun County, Yunnan Province, 202404BI090006, Research on Technological Innovation and Application of *Lanxangia tsaoko* Industry in Nujiang River Region, 202202AE090035.

## Conflicts of Interest

The authors declare no conflicts of interest.

## Supporting information


**Figure S1:** Pictures of three fruit shapes.
**Figure S2:** Electronic nose response curve of PCS, ES, and LFS.
**Figure S3:** Pearson correlation heat map between phenotype and physiology.
**Figure S4:** OPLS‐DA model results. (A, B) OPLS‐DA score plots and permutation test plots for PCS vs. ES, PCS vs. LFS, and ES vs. LFS, respectively.
**Figure S5:** Results of broad‐targeted metabolomics. (A) Classification of common differential metabolites of three fruit shapes. (B) Clustered cluster elbow graph. The *x*‐axis is the number of clusters and the *y*‐axis is an evaluation metric for the clustering result, reflects the average variation within the cluster, the smaller the better. The figure shows that from the fourth onwards, *y*‐axis no longer decreases significantly as the number of clustered clusters increases, so the choice of four clustering. (C) Graph of molecular expression patterns obtained from Kmeans analysis. (D) Differential metabolite KEGG pathway bubble map. (E) Mantel test correlation heat map.
**Table S1:** Geographic information on three fruit shapes.
**Table S2:** Phenotype and physiological indexes of three fruit shapes.
**Table S3:** Correspondence information for 100 different non‐volatile substances.
**Table S4:** Evaluation of antioxidant capacity of different fruit shapes.
**Table S5:** Bands with strong correlation between non‐volatiles and volatiles (Deng et al. 2024; Drees et al. 2023; He, Shi, et al. 2024; He, Yang, and Wang 2024; Nallan Chakravartula et al. 2022; Yu et al. 2024; Zheng et al. 2023).

**Appendix S1:** Differences in physical and chemical index experimental details, fruit shape in phenotype, physiology, volatile/non‐volatile metabolites, and antioxidant activity.

**Appendix S2:** Contains information on all metabolites detected in the widely targeted metabolomics analysis.

**Appendix S3:** Primarily includes specific results of correlation analyses, covering antioxidant activity with metabolites, phenotype with physicochemical and chemical components, volatile substances with non‐volatile substances, non‐volatile substances with spectral features, volatile substances with spectral features, and electronic nose with spectral features.

## Data Availability

The data that supports the findings of this study are available in the [Link], [Link], [Link] of this article.
