# Supplementary material for: Comparison of Different Fruit Shapes of Lanxangia tsaoko: Differences and Relevance of Phenotypes, Antioxidant Activities, and Physicochemical Information
Source: Food Sci Nutr. 2026 Jun 30;14(7):e71993. doi: 10.1002/fsn3.71993 (PMC13316455; doi:10.1002/fsn3.71993)
Supplement: Supplementary file 1 — Figure S1: Pictures of three fruit shapes. Figure S2: Electronic nose response curve of PCS, ES, and LFS. Figure S3: Pearson correlation heat map between phenotype and physiology. Figure S4: OPLS‐DA model results. (A, B) OPLS‐DA score plots and permutation test plots for PCS vs. ES, PCS vs. LFS, and ES vs. LFS, respectively. Figure S5: Results of broad‐targeted metabolomics. (A) Classification of common differential metabolites of three fruit shapes. (B) Clustered cluster elbow graph. The x‐axis is the number of clusters and the y‐axis is an evaluation metric for the clustering result, reflects the average variation within the cluster, the smaller the better. The figure shows that from the fourth onwards, y‐axis no longer decreases significantly as the number of clustered clusters increases, so the choice of four clustering. (C) Graph of molecular expression patterns obtained from Kmeans analysis. (D) Differential metabolite KEGG pathway bubble map. (E) Mantel test correlation heat map. Table S1: Geographic information on three fruit shapes. Table S2: Phenotype and physiological indexes of three fruit shapes. Table S3: Correspondence information for 100 different non‐volatile substances. Table S4: Evaluation of antioxidant capacity of different fruit shapes. Table S5: Bands with strong correlation between non‐volatiles and volatiles (Deng et al. 2024; Drees et al. 2023; He, Shi, et al. 2024; He, Yang, and Wang 2024; Nallan Chakravartula et al. 2022; Yu et al. 2024; Zheng et al. 2023). [file FSN3-14-e71993-s001.docx]

**Comparison of different fruit shapes of *Lanxangia tsaoko*: differences and relevance of phenotypes, antioxidant activities, physicochemical information**

Dengke Fu^a, b^, Tianmei Yang^a^, Weize Yang^a^, Zongliang Xu^a^, Meiquan Yang^a^, Yuanzhong Wang^a,*^, Jinyu Zhang^a,*^

^a^ Medicinal Plants Research Institute, Yunnan Academy of Agricultural Sciences, Kunming 650200, China.

^b^ College of Traditional Chinese Medicine, Yunnan University of Chinese Medicine, Yunnan 650500, Kunming, China.

* Corresponding author: Yuanzhong Wang & Jinyu Zhang

1. **mail:**

Dengke Fu: [1301860527@qq.com;](mailto:1301860527@qq.com;)

Tianmei Yang: [182552761@qq.com;](mailto:182552761@qq.com;)

Weize Yang: [yangweize116@163.com;](mailto:yangweize116@163.com;)

Zongliang Xu: [252319823@qq.com;](mailto:252319823@qq.com;)

Meiquan Yang: [158364106@qq.com;](mailto:158364106@qq.com;)

Yuanzhong Wang: [boletus@126.com;](mailto:boletus@126.com;)

Jinyu Zhang: jyzhang2015@126.com.

**Measurement of physiological indicators and antioxidant activity**

**Figures and tables**

Determination of soluble sugars

Determination of soluble proteins

Determination of soluble solid

Determination of total phenols

Determination of titratable acid

Determination of ash content

DPPH radical scavenging ability determination

Determination of the free radical scavenging ability of ABTS

Determination of FRAP

**Figures**

Fig. S1. Pictures of three fruit shapes

Fig S2. Electronic nose response curve of PCS, ES and LFS.

Fig. S3. Pearson correlation heat map between phenotype and physiology.

Fig. S4. OPLS-DA model results.

**Tables**

Table S1. Geographic information on three fruit shapes.

Table S2. Phenotype and physiological indexes of three fruit shapes.

Table S3. Correspondence information for 100 different non-volatile substances.

Table S4. Evaluation of antioxidant capacity of different fruit shapes.

Table S5. Bands with strong correlation between non-volatiles and volatiles.

**Determination of soluble sugars**

First, take 0.2g of the sample and add it to a 2ml centrifuge tube. Then, add 0.8ml of distilled water. Homogenize the mixture at high speed in a high-throughput grinder. Afterwards, add 0.4ml of hydrochloric acid, and heat it in a water bath at 80℃ for 10 minutes. After the heating is completed, use a large dragon single-channel pipette gun (20μl, 200μl, 1000μl, 5ml, 10ml) to add 20μl of methyl red indicator. Use sodium hydroxide solution to adjust it to orange color, mix well, and then centrifuge at 10000g in an Eppendorf 5424 centrifuge for 10 minutes. Take the supernatant as the sample solution. Prepare different concentrations of glucose standard solutions. Use a large dragon eight-channel pipette gun (5-50μl, 50-300μl) to respectively take 50μl of different concentrations of glucose standard solutions and the sample solution and add them to 1.5ml centrifuge tubes. Then, add 150μl of DNS solution through the large dragon eight-channel pipette gun. Heat it in boiling water for 5 minutes, cool it to room temperature, and add 0.8ml of distilled water and mix well. Finally, take 0.2ml of the reaction solution and place it on the enzyme strip plate of ThermoFisher MK3 microplate reader. Perform a 540nm colorimetric test. The calculation formula is as follows:

$$mg/g=\frac{C\times V\times A}{V_{1}\times M}\times0.001$$

Among them, C represents the protein content (μg) of the sample extract as determined by the standard curve; V represents the volume of the sample extraction solution (ml); M represents the weight of the sample (g); V1 represents the volume (ml) of the sample taken during the measurement process. A represents the dilution factor (Fu et al., 2025).

**Determination of soluble proteins**

We weighed 0.2 g of each of the 9 samples and added them to 2 ml centrifuge tubes. Then, using the large dragon single-channel pipette (20 μl, 200 μl, 1000 μl, 5 ml, 10 ml), we added 1 ml of phosphate buffer solution. We homogenized the samples at high speed in a high-throughput grinder and then centrifuged them for 10 minutes in an Eppendorf 5424 centrifuge. The supernatant was taken as the sample to be tested. Subsequently, we prepared standard solutions of bovine serum albumin at different concentrations. Using the large dragon eight-channel pipette (5 - 50 μl, 50 - 300 μl), we respectively aspirated different concentrations of bovine serum albumin standard solutions, the sample to be tested, 200 μl of reaction Mix reagent, and 40 μl of distilled water onto the microplate of the ThermoFisher MK3 microplate reader. We incubated it in an air bath at 60°C for 30 minutes and measured the absorbance at 562 nm. The calculation formula is as follows:

$$mg/g=\frac{C\times V\times A}{V_{1}\times M}\times0.001$$

Among them, C represents the protein content (μg) of the sample extract as determined by the standard curve; V represents the volume of the sample extraction solution (ml); M represents the weight of the sample (g); V1 represents the volume (ml) of the sample taken during the measurement process. A represents the dilution factor (Fu et al., 2025).

**Determination of** **soluble solid**

At 20℃, the refractive index of the sample solution was measured using a refractometer, and the content of soluble solids was directly read from the scale of the instrument. Each sample was precisely weighed at 0.01g, placed in a weighed beaker, and 5-10 times the volume of distilled water was added. The beaker was then placed in a boiling water bath for 30 minutes, and stirred occasionally with a glass rod. The beaker was removed, cooled to room temperature, weighed, and accurate to 0.01 g. The sample was filtered. The refractometer reading was calibrated with distilled water, and the soluble solids content was adjusted to 0% at 20℃. When the temperature was not 20℃, the correction values in the table were used for calibration. After drying the prism surface, 2-3 drops of the sample solution to be tested were added to the center of the prism, and the upper and lower prisms were immediately closed, aligned with the light source, and the color adjustment knob was turned to make the field of view divided into two parts of light and dark. Then, the prism knob was turned to make the dividing line of light and dark exactly at the crosshair of the objective lens. The percentage indicated on the scale was read and recorded along with the temperature at the time of measurement. For the undiluted sample, the reading of the refractometer indicates the content of soluble solids. For the diluted sample, the content of soluble solids is calculated using the following formula.

$$\left( \begin{matrix} \% \end{matrix} \right)=\frac{\mathfrak{p}\times\mathfrak{m}_{1}}{\mathfrak{m}_{0}}$$

P is used to measure the content of soluble solids in the solution (%, m/m); m_0_ represents the mass of the sample before dilution (g); m1 represents the mass of the diluted sample (g) (Park et al., 2025).

**Determination of** **total phenols**

Polyphenols react specifically with the FC reagent. The reaction products have the maximum absorption at a specific wavelength, and the absorbance value has a linear relationship with the polyphenol content within a certain concentration range. Take 0.1 g of the sample, wash it with 20 mL of distilled water and add it to a 25 mL volumetric flask. Heat it in a 100℃ boiling water bath for 30 minutes, then remove it, cool it down, adjust the volume, filter it, and store the filtrate. Dilute the above filtrate to the appropriate concentration, then take 1mL of the filtrate (if the polyphenol content is too high, it can be diluted appropriately) or the one-water gallocatechin standard solution, respectively add 1mL of FC chromogenic reagent (0.2 mol/L) and 3 mL of 7.5% sodium carbonate solution, adjust the volume to 10 mL with distilled water, mix well, incubate at room temperature (30-60 minutes), measure the absorbance at 765 nm, and calculate the polyphenol content of the sample based on the dilution factor and the content calculated from the standard curve. In addition, the preparation method of the one-water gallocatechin standard solution is as follows. Weigh 0.110 g of one-water gallocatechin, dissolve it in distilled water and adjust the volume to 100mL. The mass concentration of this solution is 1000mg/L. Take 0, 1.0, 2.0, 3.0, 4.0, and 5.0 mL of this solution to 100 mL volumetric flasks, adjust the volume with distilled water to the mark, and the mass concentrations of the one-water gallocatechin standard solutions obtained are 0, 10, 20, 30, 40, and 50 mg/L respectively. The content can be calculated using the following formula:

$$\left( mg/g \right)=\frac{C\times N\times V_{t}}{V_{s}\times m}\times0.001$$

C represents the content of polyphenols (μg) obtained from the standard curve; N represents the dilution factor; Vt represents the total volume of the extract (ml); The volume of liquid for the Vs test (ml); m represents the weight of the sample (Ramírez-Brewer et al., 2024).

**Determination of** **titratable acid**

Put the three types of fruit powder into a high-speed tissue grinder, homogenize, take 0.5 g of the homogenized sample, accurate to 0.0001 g, wash it with 20 mL of water and transfer it into a 50 mL volumetric flask. Place it in a water bath at 75-80℃ for 30 minutes. Shake it several times during this period. Take it out, cool it down, add water to the mark, shake and filter. According to the predicted acidity, use a pipette to draw 20 mL of the sample solution, add 2-3 drops of phenolphthalein indicator, titrate with sodium hydroxide standard solution until a slight red color appears and does not fade within 30 seconds as the endpoint. Record the volume consumed (Calamari et al., 2016).

**Determination of** **ash content**

The sample was subjected to low-temperature carbonization and high-temperature calcination to remove water and organic matter. The remaining non-combustible part consists of oxides of elements such as ash. After weighing, the crude ash content can be calculated. The numbered porcelain crucible was heated in a high-temperature electric furnace at 550 ± 25°C for 30 minutes. It was then moved to the door of the furnace to cool slightly, placed in a desiccator to cool to room temperature, and accurately weighed. The heating was repeated until the difference in weight between the two measurements did not exceed 0.5 mg, indicating a constant weight (m_0_). In the crucible with a known weight, 2-3 g of ground and dried plant samples (m_1_) were weighed and evenly distributed in the crucible. 1-2 mL of ethanol solution or 240 g/L magnesium acetate solution (to promote uniform ashing of the samples) was added to moisten the samples. Then, the crucible was placed on a temperature-regulating electric furnace, with the crucible cover tilted. The temperature of the electric furnace was adjusted to slowly heat for carbonization, until the smoke disappeared. The crucible was then moved to the high-temperature electric furnace and heated to 550 ± 25°C for about 1 hour (for soy products, meat products, egg products, aquatic products and dairy products, the heating time is 4 hours). The ash was nearly white when the carbonization was complete. The crucible was moved to the door of the furnace, cooled to below 200°C, and then placed in the desiccator to cool to room temperature and weighed. Subsequently, the crucible was heated again for 30 minutes, cooled, and weighed. This process was repeated until the difference in weight between the two measurements did not exceed 0.5 mg, indicating a constant weight (m_2_) (He et al., 2023). The content is calculated by the following formula:

%=m_2_/m_1_×100%

**DPPH radical scavenging ability determination**

Take 0.1 g of the sample, add 1 mL of the extraction solution, and extract using a tissue homogenizer in an ice bath. Centrifuge at 8000g for 10 minutes at 4℃. Take the supernatant and place it on ice for measurement. Then, preheat the spectrophotometer for more than 30 minutes, adjust the wavelength to 515 nm, and zero the instrument with absolute ethanol. Prepare 10mg/mL vitamin C solution using the extraction solution to make 1, 0.5, 0.25, 0.13, 0.0625, 0.03125, and 0.015625 mg/mL vitamin C solutions for later use. Add the reagents according to the table below:

| Reagent Name (μl) | Blank tube | Testing tube | Control valve | Positive control tube |
| --- | --- | --- | --- | --- |
| The supernatant |  | 50 | 50 |  |
| Reagent 3 |  |  |  | 50 |
| Extract solution | 50 |  |  |  |
| Reagent 1 |  |  | 950 |  |
| Work fluid | 950 | 950 |  | 950 |

Vortex mixing, incubation at room temperature in the dark for 30 minutes, and measurement of absorbance at 515 nm. These values are recorded as A blank, A determination, A control, and A positive control. Each determination tube should have a control tube. The positive control standard curve and the blank tube only need to be measured 1-2 times.

Reagent 1: Anhydrous ethanol is provided by the user.

Reagent 2: The powder is placed in the EP tube inside the bottle. Before use, add 4.05 mL of Reagent 1 and shake to dissolve. The unused reagent can be stored at -20°C for 1 month. It is recommended to store it in aliquots to avoid repeated freezing and thawing. Before use, prepare the working solution according to the ratio of Reagent 2 : Reagent 1 (V:V) = 4 : 21 based on the required amount for the test. Use it immediately. The unused working solution can be stored at 2-8°C for one week

Reagent 3: 10 mg of Vitamin C. Before use, add 1mL of the extraction solution and shake thoroughly to dissolve it. Prepare a 10 mg/mL solution of Vitamin C. Store at 2-8℃ for two weeks; used as a positive control (Yu et al., 2025).

The DPPH stock solution in this experiment was prepared by dissolving the dry powder in anhydrous ethanol. Before use, the working solution was prepared at a ratio of stock solution to anhydrous ethanol of 4:21, with a final reaction system concentration of 0.1 mmol/L. The preparation and storage strictly followed the kit instructions and literature methods, ensuring standardized procedures and reliable results.

The formula for calculating the free radical scavenging rate of the sample:

DPPH free radical scavenging rate D% = [[A blank - (A measurement - A control)] ÷ A blank] × 100%

**Determination of the free radical scavenging ability of ABTS**

Allow the spectrophotometer/enzyme analyzer to preheat for more than 30 minutes. Adjust the wavelength to 405 nm. Zero the spectrophotometer with distilled water. Preparation of positive control: If a linear relationship is required, it is recommended to prepare 1.5, 1.2, 0.9, 0.6, 0.3, and 0.15 mmol/L vitamin C solutions from the extract using a 10 mmol/L vitamin C solution for later use.

Operation Table: Add the following reagents to the 96-well plate or EP tubes respectively.

| Reagent Name (μl) | Blank tube | Testing tube | Control valve | Positive control tube |
| --- | --- | --- | --- | --- |
| The supernatant |  | 20 | 20 |  |
| Different concentrations of VC solution |  |  |  | 20 |
| Distilled water | 20 |  |  |  |
| Reagent Four working solution | 20 | 20 |  | 20 |
| ABTS solution | 160 | 160 |  | 160 |
| Reagent 1 |  |  | 180 |  |

Mix thoroughly, place at room temperature and shield from light for 6 minutes. Measure the absorbance at 405nm. Record them as A_blank, A_test, A_control, A_positive_control. The positive control standard curve and the blank tube only need to be measured 1-2 times.

Reagent 2: Before use, add 1 mL of distilled water and stir to fully dissolve; any unused reagent can be aliquoted for storage. It can be preserved at -20°C for four weeks. Avoid repeated freezing and thawing.

Preparation of the working solution of Reagent 3: Place the liquid in an EP tube inside the reagent bottle. Before use, prepare the Reagent Three working solution according to the ratio of Reagent Three (μL): Distilled Water (mL) = 1 μL: 12 mL based on the sample volume. Prepare it as needed and use it up within 4 hours.

Preparation of the working solution of reagent four: You can first store reagent four at -20℃ in separate portions. Before use, prepare the working solution of reagent four according to the ratio of reagent four to reagent one (V:V) = 1:9 based on the sample volume. Use it immediately and, if there is any unused reagent, store it at -20℃ for two weeks.

Reagent 5: The powder is placed in a glass bottle of the reagent. It contains 5mg of vitamin C. Before use, add 2.8mL of the extraction solution and shake thoroughly to dissolve; prepare a 10mmol/L vitamin C solution for the positive control. It can be stored at 2-8℃ for two weeks.

Preparation of ABTS working solution: Before use, prepare the ABTS working solution according to the required amount for the experiment in the ratio of reagent one : reagent two : reagent three working solution (V:V:V) = 76:5:4. Prepare it as needed and store it at room temperature in the dark. It must be used within 30 minutes (Yu et al., 2025).

The formula for calculating the free radical scavenging rate of the sample:

ABTS free radical scavenging rate D% = [(A blank - (A measurement - A control)) ÷ A blank] × 100%

**Determination of FRAP**

Weigh 0.1 g of the sample, add 1 mL of the extraction solution, and extract using a tissue homogenizer in an ice bath. Centrifuge at 8000 g for 4℃ for 10 minutes. Take the supernatant and place it on ice for measurement. Allow the enzyme detector to preheat for more than 30 minutes. Adjust the wavelength to 593 nm and zero the instrument with distilled water. The 100 μmol/mL standard solution was diluted successively with double-distilled water to 0.15, 0.3, 0.6, 0.9, 1.2, and 1.5 μmol/mL.

| μl | Measurement tube | Blank tube | Standard tube |
| --- | --- | --- | --- |
| Sample | 20 |  |  |
| Extract solution |  | 20 |  |
| Standard sample |  |  | 20 |
| Work fluid | 180 | 180 | 180 |

In this study, the "working solution" referred to in the original text is the FRAP working reagent. Each tube in the experiment was added with 180 μL of this reagent, incubated at 37°C in the dark for 30 minutes, and then the absorbance was measured at 450 nm. The antioxidant capacity of the samples was quantified by combining with the Fe^2+^ standard curve. The method complies with general specifications, and the results are reliable. Plot a standard curve with the absorbance value as the abscissa and different concentrations of standard samples as the ordinate. x represents the absorbance value, and y represents the Fe^2+^ concentration (μmol/mL) (Yu et al., 2025).

$$FRAP\left( \mu molFe2+/mL \right)=y\times V_{1}\div\left( W\times V_{1}\div V_{T} \right)$$

V_1_: Add sample volume, 0.02 mL

V_T_: Add the volume of the extracted liquid, 1 mL

W: Sample quality, g


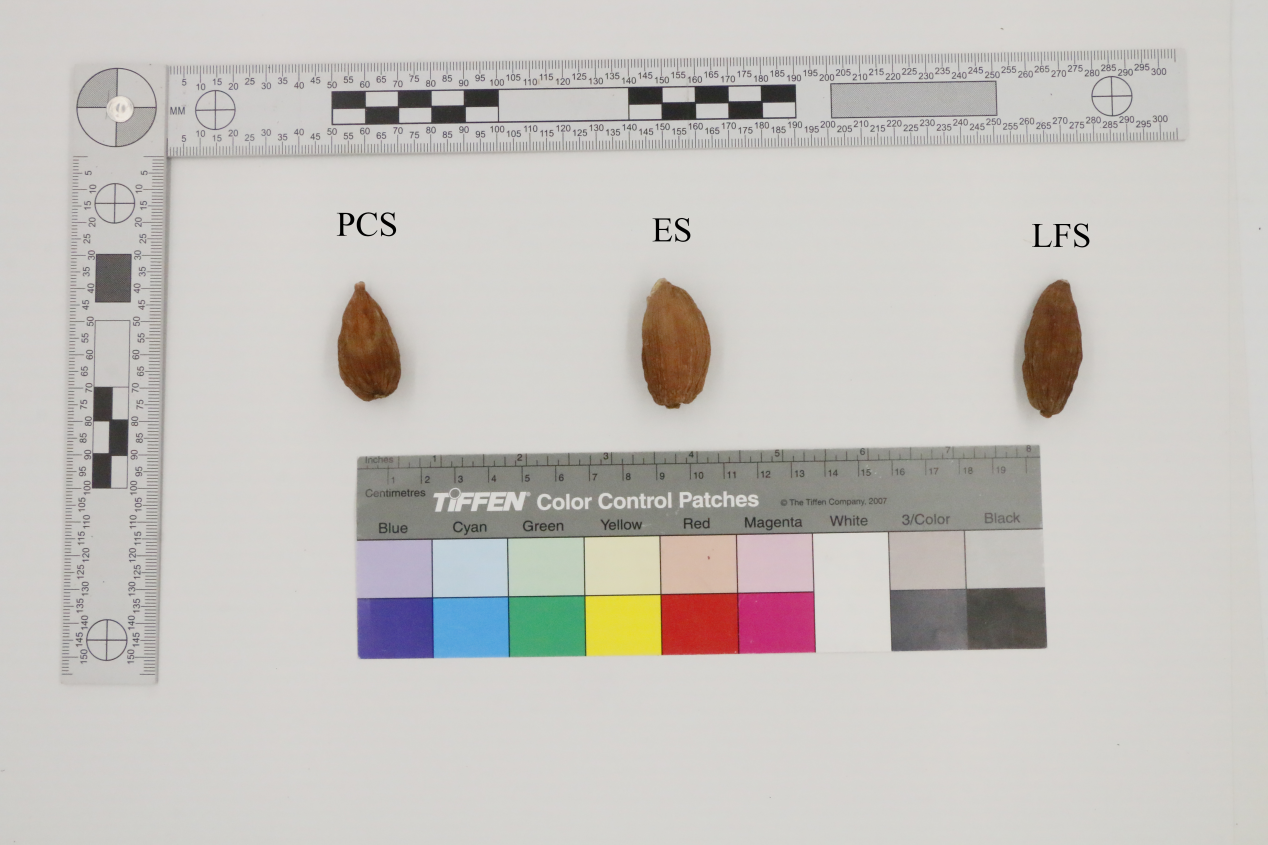


**Fig. S1.** Pictures of three fruit shapes.


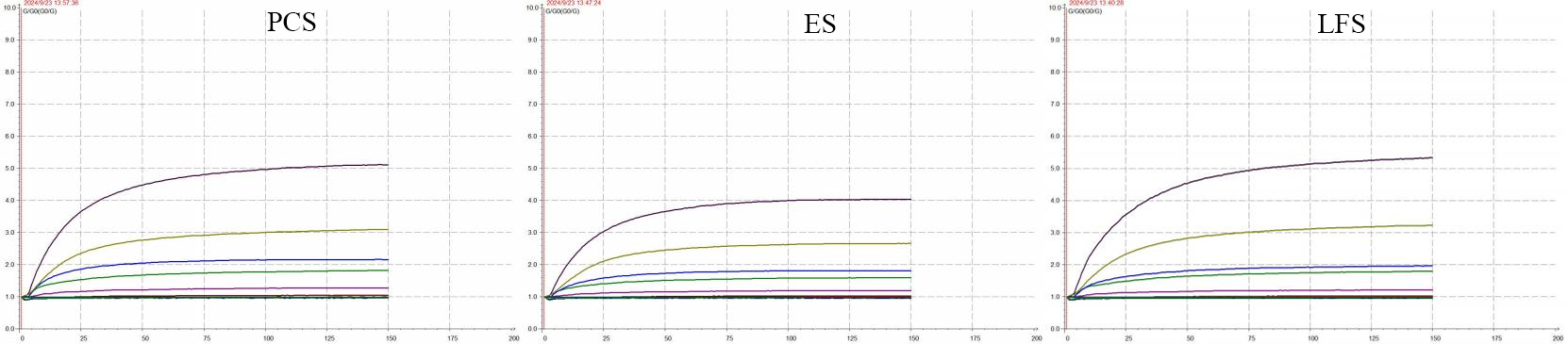


**Fig S2.** Electronic nose response curve of PCS, ES and LFS

**
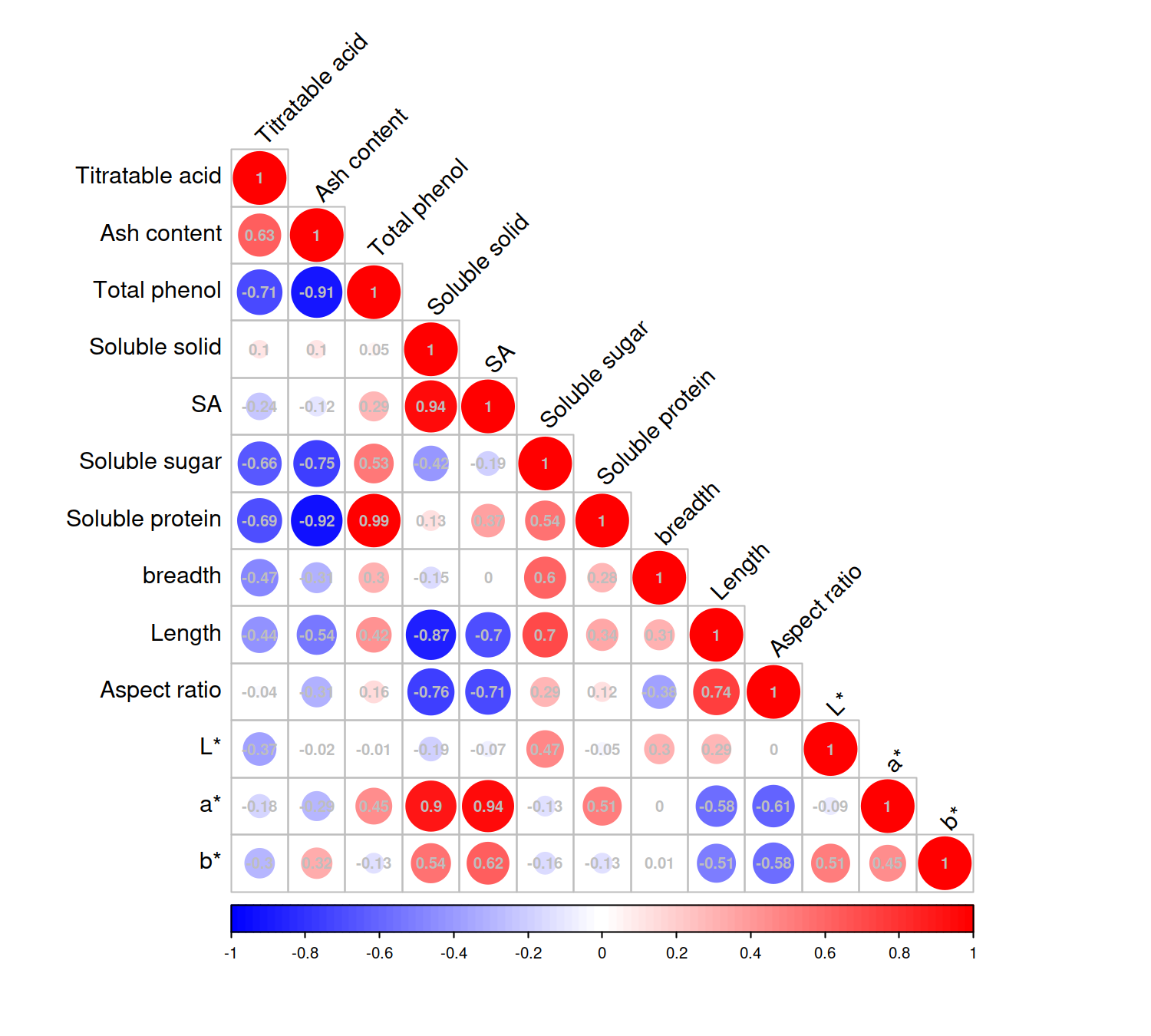
**

**Fig. S3. Pearson correlation heat map between phenotype and physiology.**


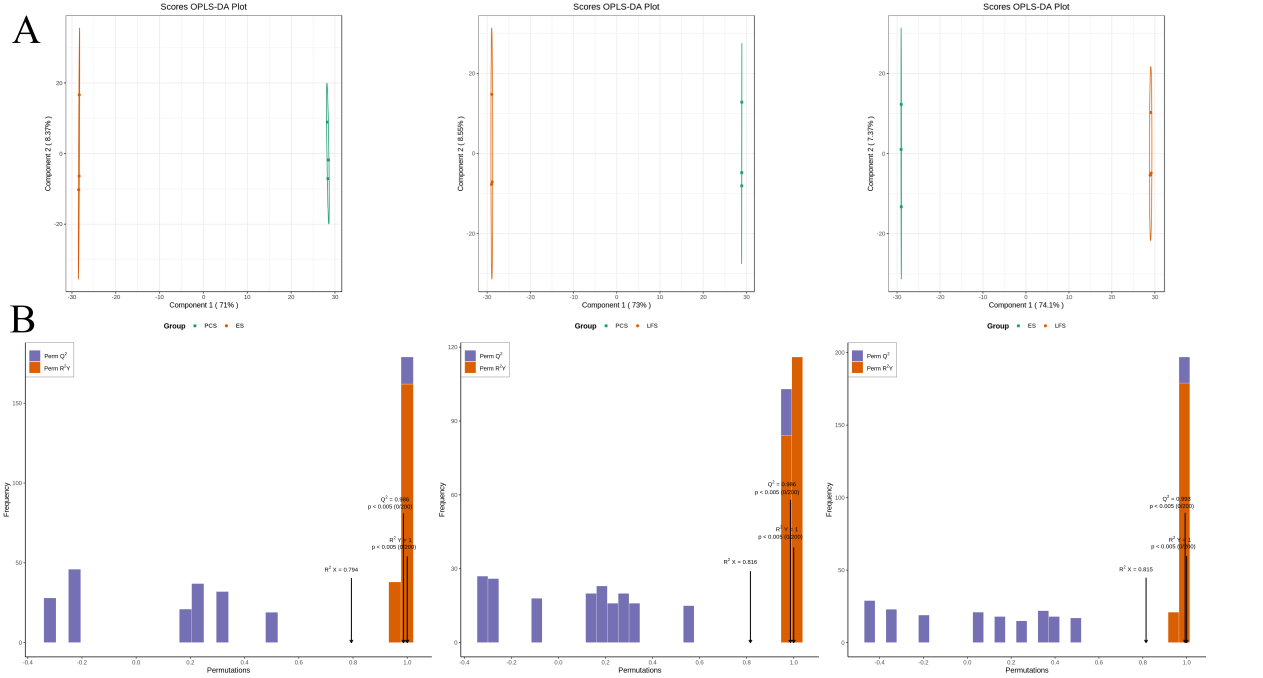


**Fig. S4.** OPLS-DA model results. **A**, **B**, OPLS-DA score plots and permutation test plots for PCS vs. ES, PCS vs. LFS, and ES vs. LFS, respectively.


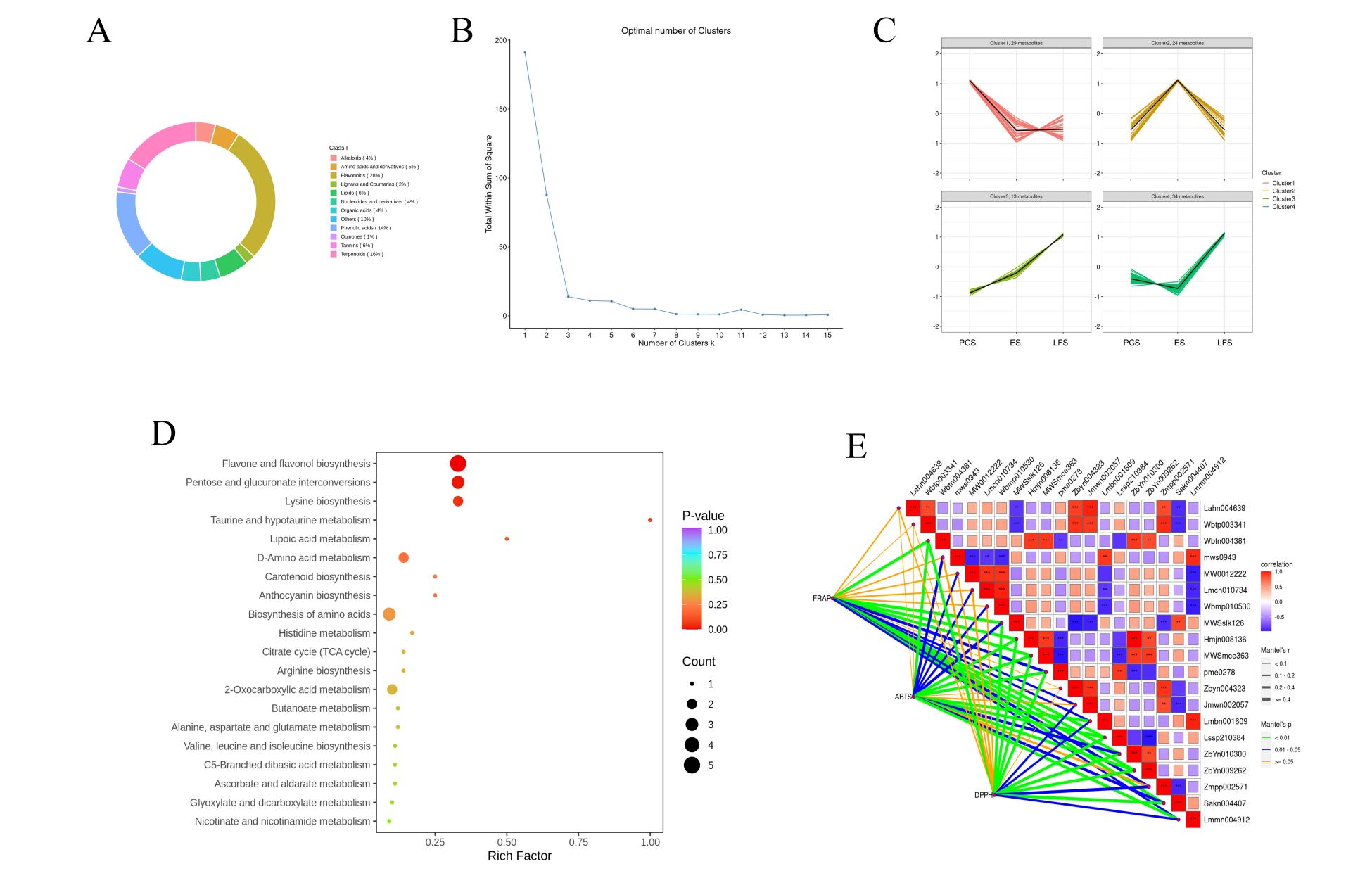


**Fig. S5.** Results of broad-targeted metabolomics. **A**, classification of common differential metabolites of three fruit shapes; **B**, clustered cluster elbow graph. The x-axis is the number of clusters and the y-axis is an evaluation metric for the clustering result, reflects the average variation within the cluster, the smaller the better. The figure shows that from the fourth onwards, y-axis no longer decreases significantly as the number of clustered clusters increases, so the choice of four clustering; **C**, Graph of molecular expression patterns obtained from Kmeans analysis; **D**, Differential metabolite KEGG pathway bubble map; **E**, mantel test correlation heat map.

**Table S1**. Geographic information on three fruit shapes.

| Collection site | Longitude | Latitude | Altitude |
| --- | --- | --- | --- |
| Galabo, Yunnan Province, China | 98°67′ E | 27°74′N | 1486 m |
| Laibin Chaoyang District, Laibin City, Guangxi Province, China | 105°66′E | 23°15′N | 1442 m |
| Dengcao Village, Tiechang Township, Malipo County, Yunnan Province, China | 104°70′E | 23°13′N | 1108 m |

**Table S2.** Phenotype and physiological indexes of three fruit shapes.

| Fruit shapes | Breadth | Length | Aspect ratio | L* | a* | b* | Soluble sugar (mg  /g) | Soluble protein (mg/g) | Soluble solid (mg/g) | Titratable acid (mg/g) | SA (sugar-acid ratio) | Ash content (mg/g) | Total phenol(g/mg) |
| --- | --- | --- | --- | --- | --- | --- | --- | --- | --- | --- | --- | --- | --- |
| PCS | 16.16±2.24a | 32.60±5.87b | 2.04±0.41ab | 43.80±2.90a | 13.96±0.25b | 22.32±1.56a | 113.77±3.44a | 28.91±1.51c | 52.70±1.17a | 5.01±0.19a | 10.54±0.61a | 79.39±2.83a | 5.85±0.21c |
| ES | 17.73±2.00a | 34.50±3.09ab | 1.95±0.19b | 42.43±1.76a | 12.48±0.30c | 20.16±0.85b | 121.65±5.35a | 37.21±2.67b | 51.57±1.16a | 4.37±0.34b | 11.86±0.94a | 66.39±2.17c | 8.27±0.37a |
| LFS | 16.60±3.14a | 37.74±4.32a | 2.30±0.27a | 43.43±1.60a | 14.66±0.31a | 21.99±1.03a | 121.97±1.32a | 53.39±2.25a | 30.69±1.04b | 4.63±0.12ab | 6.63±0.21b | 72.55±1.10b | 6.85±0.24b |

Note: Data are expressed as mean ± SD, lower case letters indicate significant differences in duncan's multiple range test (*P* < 0.05). The aspect ratio is the length and breadth of the fruits. SA is the ratio of soluble solid to titratable acid.

**Table S3.** Correspondence information for 100 different non-volatile substances.

| Q1 (Da) | Q3 (Da) | Molecular weight (Da) | Formula | Ionization model | Compounds | Class I | Class II | CAS | cpd_ID |
| --- | --- | --- | --- | --- | --- | --- | --- | --- | --- |
| 319.0441 | 183.0297 | 320.0532 | C15H12O8 | [M-H]- | 1,3,6,8-tetrahydroxy-2,5-dimethoxyxanthen-9-one | Flavonoids | Other Flavonoids | - | - |
| 255.1007 | 121.0278 | 254.0943 | C16H14O3 | [M+H]+ | 2'-Hydroxy-2-methoxychalcone | Flavonoids | Chalcones | 42220-77-9 | - |
| 303.05 | 257.05 | 302.0427 | C15H10O7 | [M+H]+ | 3,3',4',5',7-Pentahydroxyflavone | Flavonoids | Flavonols | 490-31-3 | C10177 |
| 315.051 | 271.0195 | 316.0583 | C16H12O7 | [M-H]- | 3-O-Methylquercetin | Flavonoids | Flavonols | 1486-70-0 | C04443 |
| 301.0717 | 286.0542 | 300.0634 | C16H12O6 | [M+H]+ | 6,7,8-Tetrahydroxy-5-methoxyflavone | Flavonoids | Flavones | - | - |
| 627.16 | 303.05 | 626.1483 | C27H30O17 | [M+H]+ | 6-Hydroxykaempferol-6,7-*O*-Diglucoside | Flavonoids | Flavonols | - | - |
| 435.0922 | 303.0505 | 434.0849 | C20H18O11 | [M+H]+ | Quercetin-3-*O*-α-L-arabinofuranoside | Flavonoids | Flavonols | 572-30-5 | C22608 |
| 301.0707 | 286.0798 | 300.0634 | C16H12O6 | [M+H]+ | Chrysoeriol | Flavonoids | Flavones | 491-71-4 | C04293 |
| 451.12 | 289.07 | 450.1162 | C21H22O11 | [M+H]+ | Eriodictyol-3'-*O*-glucoside | Flavonoids | Flavanones | - | - |
| 595.17 | 287.06 | 596.1741 | C27H32O15 | [M-H]- | Eriodictyol-7-*O*-Rutinoside | Flavonoids | Flavanones | 13463-28-0 | C09732 |
| 307.0812 | 139.0387 | 306.074 | C15H14O7 | [M+H]+ | Gallocatechin | Flavonoids | Flavanols | 970-73-0 | C12127 |
| 451.12 | 289.07 | 450.1162 | C21H22O11 | [M+H]+ | Maesopsin 4-*O*-Glucoside | Flavonoids | Other Flavonoids | - | - |
| 433.08 | 300.03 | 434.0849 | C20H18O11 | [M-H]- | Morin-3-*O*-lyxoside | Flavonoids | Flavonols | - | - |
| 435.0922 | 303.0522 | 434.0849 | C20H18O11 | [M+H]+ | Morin-3-*O*-xyloside | Flavonoids | Flavonols | - | - |
| 315.0497 | 300.0279 | 316.0583 | C16H12O7 | [M-H]- | Pedalitin | Flavonoids | Flavones | 22384-63-0 | C10119 |
| 579.17 | 271.06 | 579.1708 | C27H31O14+ | [M]+ | Pelargonidin-3-*O*-rutinoside | Flavonoids | Anthocyanidins | - | C12644 |
| 757.2181 | 303.0514 | 756.2113 | C33H40O20 | [M+H]+ | Quercetin-3-*O*-(2''-*O*-Rhamnosyl)rutinoside | Flavonoids | Flavonols | 55696-57-6 | - |
| 611.16 | 303.05 | 610.1528 | C27H30O16 | [M+H]+ | Quercetin-3-*O*-(2''-*O*-rhamnosyl)galactoside | Flavonoids | Flavonols | - | - |
| 549.0886 | 300.0322 | 550.0959 | C24H22O15 | [M-H]- | Quercetin-3-*O*-(6''-*O*-malonyl)glucoside | Flavonoids | Flavonols | 96862-01-0 | C12638 |
| 433.08 | 300.03 | 434.0849 | C20H18O11 | [M-H]- | Quercetin-3-*O*-arabinoside | Flavonoids | Flavonols | 22255-13-6 | - |
| 465.1 | 303.06 | 464.0955 | C21H20O12 | [M+H]+ | Quercetin-3-*O*-galactoside | Flavonoids | Flavonols | 482-36-0 | C10073 |
| 465.1 | 303.05 | 464.0955 | C21H20O12 | [M+H]+ | Quercetin-3-*O*-glucoside | Flavonoids | Flavonols | 482-35-9 | C05623 |
| 449.1078 | 303.0502 | 448.1006 | C21H20O11 | [M+H]+ | Quercetin-3-*O*-rhamnoside | Flavonoids | Flavonols | 522-12-3 | C01750 |
| 435.0922 | 303.0606 | 434.0849 | C20H18O11 | [M+H]+ | Quercetin-3-*O*-xyloside | Flavonoids | Flavonols | 549-32-6 | - |
| 463.0882 | 301.0363 | 464.0955 | C21H20O12 | [M-H]- | Quercetin-4'-*O*-glucoside | Flavonoids | Flavonols | 20229-56-5 | - |
| 465.1 | 303.05 | 464.0955 | C21H20O12 | [M+H]+ | Quercetin-5-*O*-β-D-glucoside | Flavonoids | Flavonols | - | - |
| 771.2243 | 317.065 | 770.2269 | C34H42O20 | [M+H]+ | Rhamnetin-3-*O*-Rutinoside-5-*O*-rhamnoside | Flavonoids | Flavonols | - | - |
| 451.12 | 289.07 | 452.1319 | C21H24O11 | [M-H]- | catechin-4-β-D-galactopyranoside | Flavonoids | Flavanols | - | - |
| 261.1856 | 261.1856 | 262.1933 | C17H26O2 | [M-H]- | 14,15,16-Trinorlabda-8(17),11-dien-13-oic acid | Terpenoids | Terpene | - | - |
| 317.2115 | 215.1789 | 316.2038 | C20H28O3 | [M+H]+ | 15-Hydroxydehydroabietic acid | Terpenoids | Ditepenoids | 54113-95-0 | - |
| 469.33 | 469.33 | 470.3396 | C30H46O4 | [M-H]- | 2,3-Dihydroxyoleana-11,13(18)-dien-28-oic acid | Terpenoids | Triterpene | 71850-15-2 | - |
| 177.0569 | 147.0463 | 178.063 | C10H10O3 | [M-H]- | 6,7-Dihydroxy-indan-4-carbaldehyde | Terpenoids | Monoterpenoids | - | - |
| 375.1293 | 213.0774 | 376.1371 | C16H24O10 | [M-H]- | 8-Epiloganic acid | Terpenoids | Monoterpenoids | 82509-41-9 | - |
| 317.2123 | 273.221 | 318.2195 | C20H30O3 | [M-H]- | Amomax B | Terpenoids | Ditepenoids | - | - |
| 303.2 | 179.07 | 302.1882 | C19H26O3 | [M+H]+ | Amomaxin A | Terpenoids | Ditepenoids | - | - |
| 423.1821 | 259.1333 | 424.1886 | C25H28O6 | [M-H]- | Amomutsaokin B | Terpenoids | Monoterpenoids | - | - |
| 423.1806 | 205.0494 | 424.1886 | C25H28O6 | [M-H]- | Amomutsaokin C | Terpenoids | Monoterpenoids | - | - |
| 423.1806 | 205.0494 | 424.1886 | C25H28O6 | [M-H]- | Amomutsaokin D | Terpenoids | Monoterpenoids | - | - |
| 431.12 | 269.07 | 432.1268 | C18H24O12 | [M-H]- | Asperulosidic acid | Terpenoids | Monoterpenoids | 25368-11-0 | - |
| 281.14 | 171.12 | 282.1467 | C15H22O5 | [M-H]- | Dihydrophaseic acid | Terpenoids | Sesquiterpenoids | 41756-77-8 | C15971 |
| 153.13 | 97.06 | 152.1201 | C10H16O | [M+H]+ | Fenchone | Terpenoids | Monoterpenoids | 1195-79-5 | - |
| 317.2122 | 273.2228 | 318.2195 | C20H30O3 | [M-H]- | Isocoronarin D | Terpenoids | Ditepenoids | 138965-88-5 | - |
| 303.1968 | 179.0696 | 302.1882 | C19H26O3 | [M+H]+ | Maximumin B | Terpenoids | Ditepenoids | - | - |
| 313.18 | 313.18 | 314.1882 | C20H26O3 | [M-H]- | triptobenzene N | Terpenoids | Ditepenoids | - | - |
| 329.14 | 161.06 | 330.1467 | C19H22O5 | [M-H]- | (1R,7S)-1,12,13-trihydroxybisabola-3,10-diene | Phenolic acids | Phenolic acids | - | - |
| 477.1615 | 315.11 | 478.1686 | C20H30O13 | [M-H]- | 2-(3-β-D-glucopyranosyloxy-4-hydroxyphenyl)ethanol-1-*O*-β-D-glucopyranoside | Phenolic acids | Phenolic acids | - | - |
| 771.2376 | 609.2078 | 772.2426 | C34H44O20 | [M-H]- | 3,4-Dihydroxyphenethyl alcohol-8-*O*-[4-*O*-caffeoyl-β-D-apinosyl(1→3)-β-D-glucosyl(1→6)]-β-D-glucoside | Phenolic acids | Phenolic acids | - | - |
| 589.1187 | 437.1094 | 590.1272 | C27H26O15 | [M-H]- | 4',6'-*O*-Digalloylsalicin | Phenolic acids | Phenolic acids | - | - |
| 451.0882 | 331.0677 | 452.0955 | C20H20O12 | [M-H]- | 4-Hydroxybenzoyl-1-*O*-(6''-*O*-galloyl)glucoside | Phenolic acids | Phenolic acids | - | - |
| 667.2226 | 505.17 | 668.2316 | C31H40O16 | [M-H]- | 4-Hydroxyphenethoxy-8-*O*-β-D-[6-*O*-(4-*O*-β-D-glucopyranosyl)-sinapoyl]glucopyranoside | Phenolic acids | Phenolic acids | - | - |
| 179.07 | 119.05 | 178.063 | C10H10O3 | [M+H]+ | 4-Methoxycinnamic acid | Phenolic acids | Phenolic acids | 830-09-1 | - |
| 335.08 | 179.03 | 336.0845 | C16H16O8 | [M-H]- | 5-*O*-Caffeoylshikimic acid | Phenolic acids | Phenolic acids | 73263-62-4 | - |
| 431.16 | 269.1 | 432.1632 | C19H28O11 | [M-H]- | Benzyl-β-gentiobioside | Phenolic acids | Phenolic acids | 56775-64-5 | - |
| 197.05 | 135.04 | 198.0528 | C9H10O5 | [M-H]- | Danshensu; Salvianic Acid A | Phenolic acids | Phenolic acids | 76822-21-4 | C22038 |
| 449.24 | 259.06 | 450.2101 | C20H34O11 | [M-H]- | Ginkgoside A | Phenolic acids | Phenolic acids | - | - |
| 291.2047 | 177.06 | 290.1882 | C18H26O3 | [M+H]+ | Methyl-[6]-Shogaol | Phenolic acids | Phenolic acids | - | - |
| 465.1022 | 303.0474 | 466.1111 | C21H22O12 | [M-H]- | Protocatechuic acid 1-*O*-(Glucosylvanilloyl) | Phenolic acids | Phenolic acids | - | - |
| 179.07 | 147.04 | 178.063 | C10H10O3 | [M+H]+ | p-Coumaric acid methyl ester | Phenolic acids | Phenolic acids | 19367-38-5 | - |
| 303.19 | 285.18 | 280.2038449 | C17H28O3 | [M+Na]+ | 12S-HHT | Lipids | Free fatty acids | 54397-84-1 | C20388 |
| 297.24 | 279.23 | 298.2507951 | C18H34O3 | [M-H]- | 3-Oxooctadecanoic acid | Lipids | Free fatty acids | - | - |
| 327.23 | 137.06 | 304.2402304 | C20H32O2 | [M+Na]+ | 8,11-eicosadiynoic acid | Lipids | Free fatty acids | 82073-91-4 | - |
| 291.1985 | 165.1292 | 292.2038 | C18H28O3 | [M-H]- | 9-Hydroxyoctadeca-6,10,12,15-Tetraenoic Acid | Lipids | Free fatty acids | 326492-79-9 | - |
| 313.24 | 183.14 | 314.2457 | C18H34O4 | [M-H]- | Hydroxy ricinoleic acid | Lipids | Free fatty acids | - | - |
| 311.2592 | 183.1388 | 312.2664 | C19H36O3 | [M-H]- | Ricinoleic acid methyl ester | Lipids | Free fatty acids | 141-24-2 | - |
| 577.13 | 287.06 | 576.1268 | C30H24O12 | [M+H]+ | 2α,3α-Epoxy-5,7,3',4'-tetrahydroxyflavan-(4β→8)-epicatechin | Tannins | Proanthocyanidins | - | - |
| 863.1864 | 575.1188 | 864.1902 | C45H36O18 | [M-H]- | Aesculitannin A | Tannins | Tannin | - | - |
| 867.21 | 715.2 | 866.2058 | C45H38O18 | [M+H]+ | Arecatannin C1 | Tannins | Tannin | 87727-69-3 | C17897 |
| 577.14 | 407.08 | 578.1424 | C30H26O12 | [M-H]- | Procyanidin B2 | Tannins | Proanthocyanidins | 29106-49-8 | C17639 |
| 577.14 | 407.08 | 578.1424 | C30H26O12 | [M-H]- | Procyanidin B3 | Tannins | Proanthocyanidins | 23567-23-9 | - |
| 865.2 | 577.14 | 866.2058 | C45H38O18 | [M-H]- | Procyanidin C1 | Tannins | Proanthocyanidins | 37064-30-5 | C17624 |
| 245.09 | 203.08 | 246.1004 | C13H14N2O3 | [M-H]- | N-acetyl-tryptophan | Amino acids and derivatives | Amino acids and derivatives | - | - |
| 360.14 | 191.07 | 359.1328648 | C14H21N3O8 | [M+H]+ | Pro-Glu-Asp | Amino acids and derivatives | Amino acids and derivatives | - | - |
| 316.19 | 258.14 | 315.179421 | C14H25N3O5 | [M+H]+ | Pro-Val-Thr | Amino acids and derivatives | Amino acids and derivatives | - | - |
| 425.09 | 407.08 | 426.0879052 | C13H22N4O8S2 | [M-H]- | S-Glutathionyl-L-cysteine | Amino acids and derivatives | Amino acids and derivatives | - | C05526 |
| 295.16 | 251.14 | 294.1579573 | C15H22N2O4 | [M+H]+ | Tyr-Ile | Amino acids and derivatives | Amino acids and derivatives | - | - |
| 151.0513 | 71.0128 | 152.0586 | C7H8N2O2 | [M-H]- | 1,4-Dihydro-1-Methyl-4-oxo-3-pyridinecarboxamide | Alkaloids | Pyridine alkaloids | 769-49-3 | C05843 |
| 338.122 | 176.0703 | 337.1162 | C16H19NO7 | [M+H]+ | Acetoxyindole N-Glucoside | Alkaloids | Plumerane | - | - |
| 145.1335 | 86.0961 | 144.1263 | C7H16N2O | [M+H]+ | N-Acetylcadaverine | Alkaloids | Alkaloids | 32343-73-0 | - |
| 352.16 | 220.12 | 351.1543 | C15H21N5O5 | [M+H]+ | cis-Zeatin riboside | Alkaloids | Alkaloids | 15896-46-5 | - |
| 296.0671 | 104.1078 | 295.0569 | C8H14N3O7P | [M+H]+ | 5-Aminoimidazole ribonucleotide | Nucleotides and derivatives | Nucleotides and derivatives | 25635-88-5 | - |
| 152.06 | 135.03 | 151.0494 | C5H5N5O | [M+H]+ | Guanine | Nucleotides and derivatives | Nucleotides and derivatives | 73-40-5 | C00242 |
| 366.1772 | 204.1244 | 365.1699 | C16H23N5O5 | [M+H]+ | Isopentenyladenine-7-N-glucoside | Nucleotides and derivatives | Nucleotides and derivatives | - | - |
| 352.16 | 220.12 | 351.1542689 | C15H21N5O5 | [M+H]+ | n6-(cis-hydroxyisopentenyl)adenosine | Nucleotides and derivatives | Nucleotides and derivatives | - | - |
| 191.1026 | 128.0706 | 190.0954 | C7H14N2O4 | [M+H]+ | 2,6-Diaminooimelic acid | Organic acids | Organic acids | 583-93-7 | C00666 |
| 145.0506 | 83.0503 | 146.0579 | C6H10O4 | [M-H]- | 2-Acetyl-2-Hydroxybutanoic Acid | Organic acids | Organic acids | - | C06006 |
| 137.06 | 94.04 | 136.0524295 | C8H8O2 | [M+H]+ | Phenylacetic acid | Organic acids | Organic acids | 103-82-2 | C07086 |
| 145.0142 | 101.0244 | 146.0215 | C5H6O5 | [M-H]- | α-Ketoglutaric acid | Organic acids | Organic acids | 328-50-7 | C00026 |
| 575.1018 | 423.0906 | 576.1096 | C26H24O15 | [M-H]- | 2,6-di-*O*-galloylarbutin | Lignans and Coumarins | Coumarins | - | - |
| 387.1449 | 289.0677 | 388.1522 | C21H24O7 | [M-H]- | Medioresinol | Lignans and Coumarins | Lignans | 40957-99-1 | - |
| 431.1 | 88.99 | 432.1056 | C21H20O10 | [M-H]- | kwanzoquinone C | Quinones | Anthraquinone | - | - |
| 291.0867 | 139.038 | 290.079 | C15H14O6 | [M+H]+ | (3,5-dihydroxyphenyl)-(4-hydroxy-2,6-dimethoxyphenyl)methanone | Others | Ketone compounds | - | - |
| 151.0412 | 108.0233 | 152.0473 | C8H8O3 | [M-H]- | 2,5-Dihydroxyacetophenone | Others | Ketone compounds | 490-78-8 | - |
| 639.36 | 255.23 | 580.3459 | C28H52O12 | [M+CH3COOH-H]- | 3'-Palmitoyl sucrose | Others | Saccharides | - | - |
| 661.35 | 277.22 | 602.3302 | C30H50O12 | [M+CH3COOH-H]- | 3'-linoleoyl sucrose | Others | Saccharides | - | - |
| 361.1664 | 343.1544 | 360.1573 | C20H24O6 | [M+H]+ | 5-hydroxy-1-(3,4-dihydroxyphenyl-7-(4-hydroxy-3-methoxyphenyl)-3-heptanone | Others | Ketone compounds | - | - |
| 107.05 | 77.04 | 106.0419 | C7H6O | [M+H]+ | Benzaldehyde | Others | Aldehyde compounds | 100-52-7 | C00261 |
| 195.14 | 79.05 | 194.1307 | C12H18O2 | [M+H]+ | Cnidilide | Others | Lactones | - | - |
| 431.23 | 251.17 | 432.2359 | C21H36O9 | [M-H]- | Glucosyl 10,11-dihydroxy-2,6-farneSadienoate | Others | Others | - | - |
| 151.0612 | 59.0139 | 152.0685 | C5H12O5 | [M-H]- | L-Arabitol | Others | Saccharides | 7643-75-6 | C00532 |
| 151.0612 | 59.0139 | 152.0685 | C5H12O5 | [M-H]- | Xylitol | Others | Saccharides | 87-99-0 | C00379 |

Note：Index, substance Maiwei ID; Q1 (Da), molecular weight of the parent ion of a substance after addition of ions by an electrospray ionization source; Q3 (Da), characteristic fragment ions; Molecular weight (Da), Relative molecular mass; cpd_ID, substance KEGG database number.

**Table S4.** Evaluation of antioxidant capacity of different fruit shapes.

| Fruit shape | DPPH (%) | ABTS (%) | FRAP (μmol Fe^2+^/g） |
| --- | --- | --- | --- |
| PCS | 25.61±0.68c | 63.54±0.60c | 43.68±0.53c |
| ES | 61.93±0.11a | 90.24±0.04a | 130.34±1.52a |
| LFS | 34.14±0.47b | 77.93±1.23b | 58.00±0.53b |

Note: Data are expressed as mean ± SD, lower case letters indicate significant differences in duncan's multiple range test (*P* < 0.05).

**Table S5.** Bands with strong correlation between non-volatiles and volatiles. (Deng et al., 2024; Drees et al., 2023; He et al., 2023, 2024; Nallan Chakravartula et al., 2022; Yu et al., 2024; Zheng et al., 2023)

| Composition | wave band (cm^-1^) | Vibrational modes of chemical bonds and groups |
| --- | --- | --- |
| 5-*O*-Caffeoylshikimic acid | 6129-5743 | first overtone of CH2 and CH functional groups；stretching of O–H, C–H |
|  | 7282-7247 | CH2– and CH3–third overtones |
|  | 8539-8192 | second overtone of functional groups CH, CH2, and CH3；first overtone of the O–H stretch；2nd overtone of C = C–H stretching |
|  | 10000-9372 |  |
| Quercetin-3-*O*-α-L-arabinofuranoside | 6530-5558 | first overtone of CH2 and CH functional groups；first overtone of the functional groups C=O-O, C–O, and OH；stretching of O–H, C–H ；O–H stretching |
|  | 7089-6800 | stretching of O–H, C–H |
| Quercetin-3-*O*-galactoside | 7278-5319 | first overtone of CH2 and CH functional groups；first overtone of the functional groups C=O-O, C–O, and OH；first overtone of O–H stretch in carbohydrates；first overtone of the C–H stretch for CH and –CH2；O–H stretching；first overtone O–H stretch |
| Quercetin-3-*O*-glucoside | 6430-5639 | first overtone of CH2 and CH functional groups；first overtone of the C–H stretch for CH and –CH2；O–H stretching；first overtone of C–H stretching；first overtone O–H stretch；first overtone of C–H stretching；1st overtone C–H |
| Quercetin-5-*O*-β-D-glucoside | 5928-5851 | stretching of O–H, C–H and；first overtone of the C–H stretch for CH and –CH2 |
| 2-Nonenal, (E)- | 4277-4100 | Second overtone of C–H；first overtone of –CH2 symmetric stretching；second overtones of C–H and C–H2 bending；stretching vibrations of the C–H |
| Butanal, 2-methyl- | 4435-4042 | Second overtone of C–H；first overtone of –CH2 symmetric stretching；second overtones of C–H and C–H2 bending；stretching vibrations of the C–H；stretching of C–C |
|  | 5492-5377 | stretching vibrations of the C–H |
|  | 10000-7143 | first overtone of the O–H stretch |
| Heptanal | 10000-7309 | first overtone of the O–H stretch |
| W1W | \| 10000-7517 \| \| --- \| | first overtone of the O–H stretch；C–H stretching first and second overtones |
| W2W | \| 10000-7517 \| \| --- \| | first overtone of the O–H stretch；C–H stretching first and second overtones |
| W5S | 7945-7600 | the second overtone of C–H stretching |
|  | 10000-8670 | first overtone of the O–H stretch |

**References**

2024 Application of ATR-FTIR and FT-NIR spectroscopy coupled with chemometrics for species identification and quality prediction of boletes.pdf. (n.d.).

Calamari, L., Gobbi, L., & Bani, P. (2016). Improving the prediction ability of FT-MIR spectroscopy to assess titratable acidity in cow’s milk. Food Chemistry, 192, 477–484. https://doi.org/10.1016/j.foodchem.2015.06.103

Deng, G., Li, J., Liu, H., & Wang, Y. (2024). A fast method for predicting adenosine content in porcini mushrooms using Fourier transform near-infrared spectroscopy combined with regression model. LWT, 201, 116243. https://doi.org/10.1016/j.lwt.2024.116243

Drees, A., Brockelt, J., Cvancar, L., & Fischer, M. (2023). Rapid determination of the shell content in cocoa products using FT-NIR spectroscopy and chemometrics. Talanta, 256, 124310. https://doi.org/10.1016/j.talanta.2023.124310

Fu, D.-K., Yang, W.-Z., Yang, M.-Q., Yang, T.-M., Wang, Y.-Z., & Zhang, J.-Y. (2025). Based on metabolomics and fourier transforms near infrared spectroscopy characterization of Lanxangia tsaoko chemical profile differences among fruit types and development of rapid identification and nutrient prediction models. Food Bioscience, 66, 106238. https://doi.org/10.1016/j.fbio.2025.106238

He, G., Yang, S., & Wang, Y. (2023). An integrated chemical characterization based on FT-NIR, and GC–MS for the comparative metabolite profiling of 3 species of the genus Amomum. Analytica Chimica Acta, 1280, 341869. https://doi.org/10.1016/j.aca.2023.341869

He, G., Yang, S., & Wang, Y. (2024). Suitable habitat prediction and identification of origin of Lanxangia tsao-ko. Computers and Electronics in Agriculture, 223, 109127. https://doi.org/10.1016/j.compag.2024.109127

He, H.-J., Wang, Y., Wang, Y., Liu, H., Zhang, M., & Ou, X. (2023). Simultaneous quantifying and visualizing moisture, ash and protein distribution in sweet potato [Ipomoea batatas (L.) Lam] by NIR hyperspectral imaging. Food Chemistry: X, 18, 100631. https://doi.org/10.1016/j.fochx.2023.100631

Nallan Chakravartula, S. S., Moscetti, R., Bedini, G., Nardella, M., & Massantini, R. (2022). Use of convolutional neural network (CNN) combined with FT-NIR spectroscopy to predict food adulteration: A case study on coffee. Food Control, 135, 108816. https://doi.org/10.1016/j.foodcont.2022.108816

Park, H., Eo, H. J., Kim, C.-W., Stewart, J. E., Lee, U., & Lee, J. (2025). Soluble solids content-based fruit maturity affects postharvest fruit quality by mediating metabolic alterations in cold-stored ‘Autumn Sense’ hardy kiwifruit. Food Chemistry: X, 32, 103373. https://doi.org/10.1016/j.fochx.2025.103373

Ramírez-Brewer, D., Quintana, S. E., & García-Zapateiro, L. A. (2024). Modeling and optimization of microwave-assisted extraction of total phenolics content from mango (Mangifera indica) peel using response surface methodology (RSM) and artificial neural networks (ANN). Food Chemistry: X, 22, 101420. https://doi.org/10.1016/j.fochx.2024.101420

Yu, D., Qu, C., Xu, J., Lu, J., Wu, D., & Wu, Q. (2024). Rapid discrimination and quantification of chemotypes in Perillae folium using FT-NIR spectroscopy and GC–MS combined with chemometrics. Food Chemistry: X, 24, 101881. https://doi.org/10.1016/j.fochx.2024.101881

Yu, S. M., Song, D. H., Lee, Y.-R., Kim, K. N., Kim, S.-H., & An, J. H. (2025). Chemical composition, aroma profile, antioxidant activity, and sensory attributes of beer according to roasting degree and malt type. Food Chemistry: X, 31, 103147. https://doi.org/10.1016/j.fochx.2025.103147

Zheng, C., Li, J., Liu, H., & Wang, Y. (2023). Data fusion of FT-NIR and ATR-FTIR spectra for accurate authentication of geographical indications for Gastrodia elata Blume. Food Bioscience, 56, 103308. https://doi.org/10.1016/j.fbio.2023.103308
